# Excessive Sodium Intake Leads to Cardiovascular Disease by Promoting Sex-Specific Dysfunction of Murine Heart

**DOI:** 10.3389/fnut.2022.830738

**Published:** 2022-07-01

**Authors:** Xiuli Chen, Haiying Wu, Shenzhen Huang

**Affiliations:** ^1^Obstetrical Department, Henan Provincial People's Hospital, People's Hospital of Zhengzhou University, Zhengzhou, China; ^2^Henan Eye Institute, Henan Eye Hospital and Henan Key Laboratory of Ophthalmology and Visual Science, Henan Provincial People's Hospital, People's Hospital of Henan University, People's Hospital of Zhengzhou University, Zhengzhou, China

**Keywords:** cardiovascular disease, high-salt diet, heart, transcriptome, sex-specific damage

## Abstract

**Background:**

Globally, a high-salt diet (HSD) has become a threat to human health as it can lead to a high risk of cardiac damage. Although some studies investigating HSD have been carried out, the majority has been conducted in males, and there are few female-specific studies, thereby ignoring any effects of sex-specific damage on the heart. In this study, we determined how HSD induces different pathways of cardiovascular diseases through sex-specific effects on cardiac damage in mice.

**Methods:**

An HSD murine model of male and female C57BL/6J mice was fed with sodium-rich chow (4% NaCl). After 8 weeks, cardiac tissues were collected, and the whole gene transcriptome of the hearts of male and female mice was characterized and analyzed using high-throughput RNA sequencing. Immunohistochemistry staining was used to further assess the harmful effects of HSD on protein expression of genes associated with immunity, fibrosis, and apoptosis in male and female mice.

**Results:**

HSD drastically altered the cardiac transcriptome compared to that of the normal heart in both male and female mice and had a sex-specific effect on the cardiac composition in the transcriptome. HSD produced various differentially expressed genes and affected different KEGG pathways of the transcriptome in male and female mice. Furthermore, we found that HSD induced different pathways of cardiovascular disease in the male mice and female mice. The pathway of hypertrophic cardiomyopathy is significantly enriched in HSD-treated male mice, while the pathway of dilated cardiomyopathy is significantly enriched in HSD-treated female mice. Finally, metabolism, immunity, fibrosis, and apoptosis in the mouse heart showed sex-specific changes predicting cardiac damage.

**Conclusion:**

Our results demonstrate that HSD adversely impacts cardiac structure and function by affecting the metabolism, immunity, fibrosis, and apoptosis in the murine heart and induces the mouse to suffer from sex-specific cardiovascular disease. This study provides a new perspective and basis for the differences in the pharmacology and interventional treatment of sex-specific cardiovascular diseases induced by HSD in men and women.

## Introduction

High salt content in diet poses various health risks and contributes to the increase in disease prevalence in high-income countries (1, 2). Salt is not only important for flavor in the diet, but the sodium in salt is an essential nutrient for the human body to maintain normal physiological functions. The body relies on sodium to maintain nutrient absorption, muscle movement, nerve transmission, cardiopulmonary function, and metabolism (3, 4). The World Health Organization (WHO) recommends that adults consume <5 g of iodized salt per day (5). However, the recommended daily salt intake is controversial (1, 6, 7). Studies have shown that the average salt intake in most countries is about 9–12 g/day, and many Asian countries exceed 12 g/day (8). The average salt intake of Iranians is 9.52 g/day (9), and in China, it is also >5 g/day (1). A survey by the Public Health Association of Canada revealed that more than 85% of men and 60% of women aged 19–70 years have sodium intake over the recommended amount, which leads to health risks (10). Japan is well-known for its high salt intake in diets, and existing studies have shown that the mortality rate of salt-related cardiovascular disease is high worldwide (11). Therefore, excessive sodium intake is a global problem threatening the health of many people.

Existing evidence suggests that a high-salt diet (HSD) is related to cardiovascular disease (11, 12), chronic inflammation (13), autoimmune disease (14), cognitive impairment (15), and cancer (16, 17). HSD causes elevated blood pressure and, therefore, a risk to cardiovascular function (18). Studies have shown that even if blood pressure does not rise under HSD, organ damage may still occur (18). HSD stimulates tissue remodeling of the heart and kidney (19) and induces the occurrence and development of aortic fibrosis (20). Recent studies have shown that the damage of HSD to the structure and function of organs is sex-specific (21–23). In women, HSD results in less severe damage to end organs, such as cardiac hypertrophy (CH), than in men (21). The effect of HSD on hypertension has been recognized (24, 25); however, there is a lack of understanding of the underlying mechanisms by which HSD damages heart tissue and causes cardiovascular disease in different sexes.

To investigate the effect of sex-specific damage of HSD on the structure and function of heart tissues, we generated an HSD murine model by administering male and female C57BL/6J mice with sodium-rich chow containing 4% NaCl. The whole gene transcriptome of the mouse HSD-treated heart was characterized and analyzed by high-throughput RNA sequencing. This study determined that HSD induces cardiac dysfunction and structural damage and possibly cardiovascular disease by inducing changes to metabolism, immune response, fibrosis, and apoptosis in the hearts of male and female mice. Our research may help to reveal the molecular mechanisms of physiological damage caused by HSD to cardiac tissue and is of great significance for the development of therapeutic intervention methods for HSD-induced cardiovascular-related diseases.

## Materials and Methods

### Experimental Design

**Figure 1:**
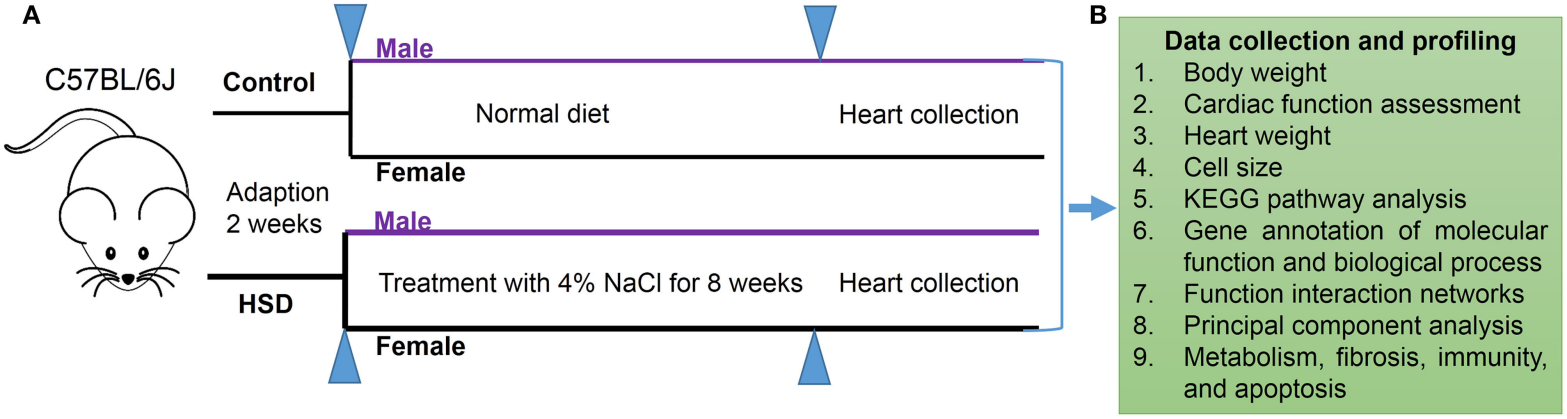
Schematic of experimental design. **(A)** Protocol of HSD for male and female C57BL/6J mice (*N* = 3 individual mice per group). **(B)** Data collection of mouse body weight, cardiac function assessment, and heart weight, and transcriptional profiling obtained from RNA-Seq by using KEGG pathway analysis, function interaction networks, and principal component analysis expression profile of metabolism, fibrosis, immunity, and apoptosis.

A schematic of the experimental design is shown in Figure 1. C57BL/6J mice of different sexes were adapted to a 12-h light/12-h dark (LD) cycle for 2 weeks and divided into a normal diet treated group and an HSD-treated group for 8 weeks. Data on mouse body weight and heart weight were collected (Figure 1A), and transcriptional profiling was obtained from RNA sequencing (RNA-Seq) using Kyoto Encyclopedia of Genes and Genomes (KEGG) pathway analysis, functional interaction networks, and principal component analysis of metabolism, fibrosis, immunity, and apoptosis expression profiles (Figure 1B).

### Animals

Twelve wild-type C57BL/6J male mice and female mice (6–8 weeks) were purchased from GemPharmatech Co., Ltd (Nanjing, Jiangsu, http://www.gempharmatech.com). Before starting the experiment, all mice were placed in a 12-h LD cycle to adapt to the environment for 2 weeks. Male and female mice were fed a normal diet and HSD (4% NaCl) as the normal and high-salt groups, respectively. All experimental protocols were approved by the Institutional Animal Care and Use Committee of Henan Province People's Hospital.

### Cardiac Function Assessment

Echocardiography of normal diet or HSD-treated male and female mice was performed in an ultrasonic echocardiographic system (Vevo2100, VisualSonics Inc., Toronto, Canada). Echocardiography of mice anesthetized with isoflurane was performed in M-mode. Fractional shortening (FS), left ventricular (LV) internal dimension at end-diastole (LVIDd), and diastolic left ventricular thickness (LVPWd) were collected.

### Tissue Collection

After 8 weeks following a normal diet or HSD, the hearts of mice were rapidly harvested and weighed after death by cervical dislocation. Tissues were immediately immersed in liquid nitrogen, transported, and stored in a freezer set to maintain −80°C.

### Immunohistochemistry of Murine Heart

Immunohistochemistry (IHC) analysis was performed on murine heart samples according to a previously published protocol (26). Paraffin-embedded heart tissues were divided into four groups: control-treated male mice (CONMH), HSD-treated male mice (MH8W), control-treated female mice (CONFH), and HSD-treated female mice (FH8W). To dewax the paraffinized samples, cardiac sections were placed in xylene I-III (10023418, Sinopharm Chemical Reagent Co., Ltd., Shanghai, China) for 15–20 min and then rinsed with 75–100% ethanol (100092683, Sinopharm Chemical Reagent Co., Ltd., Shanghai, China) for 5 min, and finally rinsed with distilled water. After using citric acid (pH 6.0, G1202, Servicebio Company, Wuhan, China), antigen retrieval solution was added to cardiac sections, then the cardiac sections were placed in 3% hydrogen peroxide solution (G0115, Servicebio Company, Wuhan, China), incubated at room temperature, protected from light for 25 min, and placed in PBS (pH 7.4, G0002, Servicebio Company, Wuhan, China). Sections were shaken and washed three times on a decolorizing shaker for 5 min each time to block the endogenous peroxidase. The cardiac sections were incubated with anti-CD68 antibody (GB113109, Servicebio Company, Wuhan, China) and anti-cleaved caspase-3 antibody (GB11009-1, Servicebio Company, Wuhan, China) overnight at 4°C. After the cardiac sections were washed with PBS on a decolorizing shaker, the secondary antibody (G23303, Servicebio Company, Wuhan, China) was added and developed with DAB (G1211, Servicebio Company, Wuhan, China), and cell nuclei were counterstained with hematoxylin (G1004, Servicebio Company, Wuhan, China). Microscopy, image acquisition, and analysis of cardiac sections were performed using a light microscope with a slide scanner (Pannoramic 250/MIDI, 3D HISTECH Ltd., Hungary). To quantify CD68 macrophage infiltration and apoptosis, we randomly selected twenty 400X field images counting the positive cells in a double-blind manner; the total cell number was counted by ImageJ software (version 1.53k, Wayne Rasband, National Institutes of Health, United States). The ratios of the number of positive cells to the total cell number were calculated by dividing the number of positive cells by the total cell number.

### Masson Staining of Murine Heart

Masson staining of murine hearts was performed according to a previously published study (27). All cardiac sections from CONMH, MH8W, CONFH, and FH8W groups were placed in xylene I-III (10023418, Sinopharm Chemical Reagent Co., Ltd., Shanghai, China) for 15–20 min and then rinsed with 75–100% ethanol (100092683, Sinopharm Chemical Reagent Co., Ltd., Shanghai, China) for 5 min and rinsed with distilled water. The cardiac sections were then stained with Masson's trichrome staining kit (G1006, Servicebio Company, Wuhan, China) according to the manufacturer's recommendations. Microscopy, image acquisition, and analysis of cardiac sections were performed using an orthotopic light microscope (Nikon ECLIPSE E100, Nikon, Japan). To quantify the degree of fibrosis, we randomly selected twenty 400X field images counting the fibrotic area in a double-blind manner; the total area was also counted by ImageJ software (version 1.53k, Wayne Rasband, National Institutes of Health, United States). The ratios of the fibrotic area to the total area were calculated by dividing the fibrotic area by the total area.

### Hematoxylin-Eosin Staining of Murine Heart

All cardiac sections from CONMH, MH8W, CONFH, and FH8W groups were placed in xylene I-III (10023418, Sinopharm Chemical Reagent Co., Ltd., Shanghai, China) for 15–20 min and then rinsed with 75–100% ethanol (100092683, Sinopharm Chemical Reagent Co., Ltd., Shanghai, China) for 5 min and rinsed with distilled water. The cardiac sections were stained with hematoxylin and eosin dye solution from a hematoxylin-eosin (HE) staining Kit (G1003, Servicebio Company, Wuhan, China). After the slices were dehydrated, they were mounted with neutral gum (10004160, Sinopharm Chemical Reagent Co., Ltd., Shanghai, China). Microscopy, image acquisition, and analysis of cardiac sections were performed using an orthotopic light microscope (Nikon ECLIPSE E100, Nikon, Japan). For quantification of cardiac cell size, 20 400X field images were randomly selected in a double-blind manner and cell numbers in each field were counted by ImageJ software (version 1.53k, Wayne Rasband, National Institutes of Health, United States). Average cell size is calculated as the number of cells in the field divided by the area of the field.

### RNA Extraction and RNA Sequencing

All RNA extractions were performed by the Beijing Genomic Institute (Shenzhen, China, http://bgitechsolutions.com/sequencing). Frozen mouse heart tissue was broken up using a mortar and pestle to obtain a piece, macroscopically free of fatty infiltration, fibrosis, and blood. RNA was extracted using the RNA Easy Spin Column Kit (Qiagen, Hilden, Germany) following the TRIzol RNA extraction protocol.

The RNA library was prepared according to a previously published study (28) using the DNBSEQ platform. Magnetic beads with OligodT tags were used to enrich mRNA with a polyA tail from total RNA. RNA was fragmented with the interrupted buffer, reverse transcription was performed using random N6 primers, and cDNA strands were synthesized to form double-stranded DNA. The synthesized double-stranded DNA ends were filled in, and the 5' end was phosphorylated to form a sticky end with a protruding “A” on the 3' end, and then a blister-like linker with a protruding “T” on the 3' end is connected. The ligation product was amplified by PCR using specific primers. The PCR product was thermally denatured, and single-stranded DNA was circularized with a bridge primer to obtain a single-stranded circular DNA library. The constructed library was quality-checked, qualified, and sequenced. The reference genome was from the species *Mus musculus*, and the version was GCF_000001635.26_GRCm38.p6, which was obtained from the National Center for Biotechnology Information (NCBI).

### Principal Component Analysis

Principal component analysis (PCA) was performed using the Statistical Analysis of Metagenomic Profiles (STAMP) software package (29). PCA was used to visualize the cluster distribution of cardiac transcripts from male and female mice in the normal group and the HSD group on PC1, PC2, and PC3. The statistical test used was ANOVA, a *post-hoc* test was set as Tykey-Kramer with 0.95, and the effect size was set as Eta-squared. The other parameters were set to default values.

### Functional Annotation With KEGG

Kyoto encyclopedia of genes and genomes pathway annotation was performed using BLASTALL software (National Center for Biotechnology Information [NCBI], Bethesda, MD, United States) against the KEGG database version 81.0 (http://www.genome.jp/kegg) and NCBI RefSeq GCF_000001635.25_GRCm38.p5 as the reference gene set. Pathways with *q* ≤ 0.05 were defined as significantly enriched in differentially expressed genes (DEGs). The *P value* formula used in this study is given as follows:


(1) $$\begin{array}{r} P=1-\overset{m-1}{\underset{i=0}{\sum }}\frac{\left(\frac{M}{i}\right)\&\left(\frac{N-M}{n-i}\right)}{\left(\frac{N}{n}\right)} \end{array}$$

where *N* is the number of genes with pathway annotations in all genes, *n* is the number of candidate genes in *N*, M is the number of genes annotated as a specific pathway in all genes, and m is the number of candidate genes annotated as a specific pathway. The *P*-value was determined using the Bonferroni correction (30), with *q* (corrected *P*-value) ≤ 0.05, as the threshold.

### Gene Ontology Enrichment Analysis

The molecular function and biological process of genes were analyzed by Gene Ontology (GO) (31, 32). Significance enrichment analysis of GO terms was performed to obtain significant relationships and biological functions of candidate genes. The analysis first maps all candidate genes to each term in the GO database (http://www.geneontology.org) and calculates the number of genes for each term. The hypergeometric test was then applied to determine the significance of the biological functions of the candidate genes in comparison to the different genetic backgrounds of this species. Referring to the software “GO:: TermFinder” (http://www.yeastgenome.org/help/analyze/go-term-finder), the calculation formula of this analysis is the same as the KEGG enrichment analysis, *N* is the number of genes with GO annotations, *n* is the number of candidate genes in *N*, and M is the number of genes annotated with a specific GO term in all genes. The calculated *P*-value was determined using the Bonferroni correction, with *q* (corrected *P-*value) ≤ 0.05 as the threshold. GO terms that met this condition were defined as significantly enriched.

### Protein-Protein Association Networks

Protein-protein association networks (PPANs) for fibrosis, metabolism, immunity, and apoptosis were analyzed using STRING (https://string-db.org/, version 11.5) (33). The network type was set as the full STRING network. The interaction sources were set as experiments and databases. The STRING network clusters were set using k-means clustering.

### Statistics and Software

GraphPad Prism software (version 8.1.0, La Jolla, CA, United States) was used to generate bar charts and scatter plots. The Venn diagram plotter, a network tool (Venny, version 2.1.0), was used to compare the number of transcripts between the normal and HSD-treated groups. The expression of transcripts in the normal and HSD groups was visualized using the pheatmap package in R (64-bit, version 3.5.3). The data were analyzed using the two-way analysis of variance (ANOVA). The data are presented as mean values ± SEM and were considered statistically significant when *P* < 0.05.

## Results

### Excessive Sodium Intake Alters the Size of Mouse Cardiomyocytes

**Figure 2:**
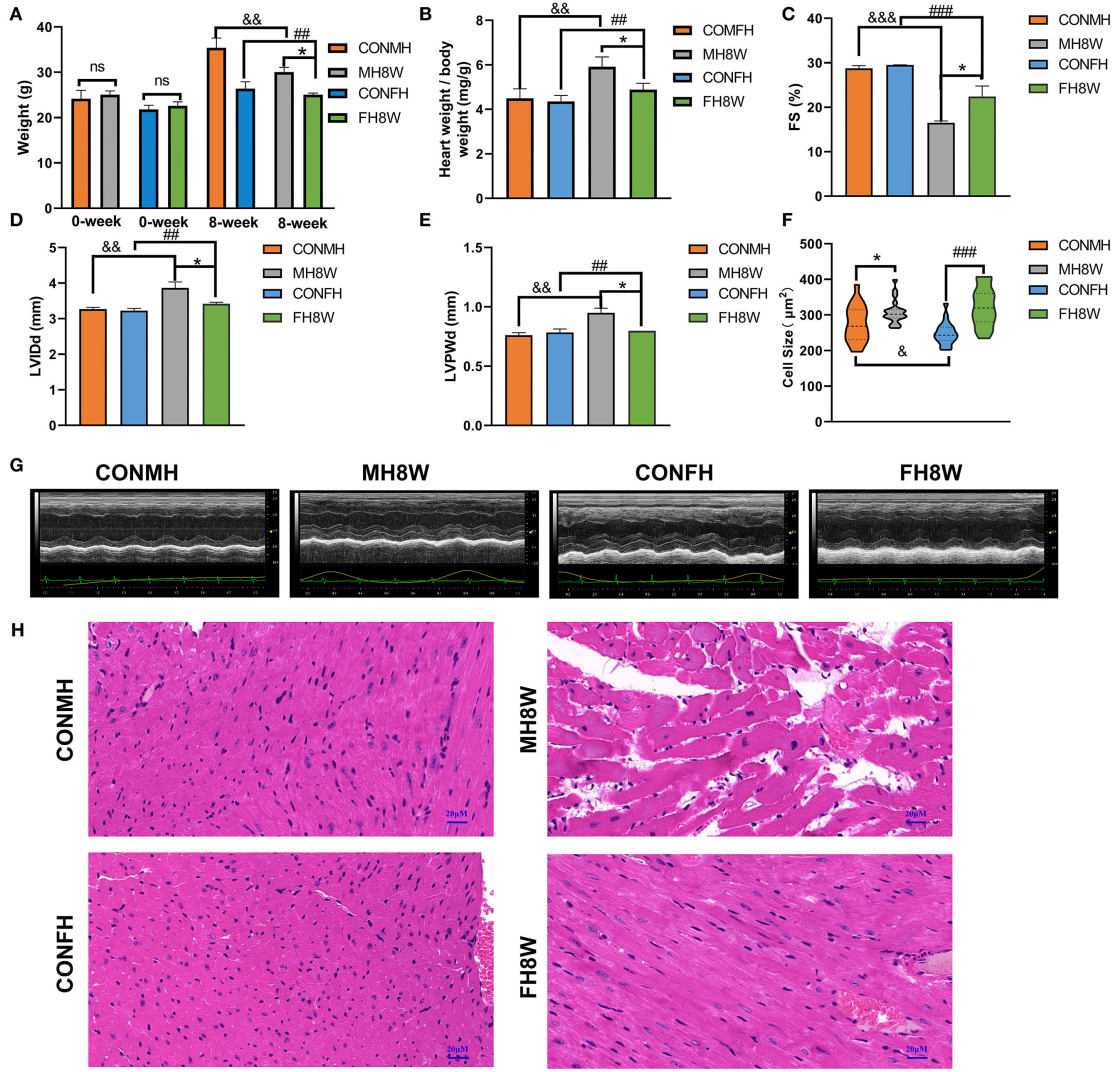
The effect of excessive sodium intake on mice. **(A)** Body weight of the control group and the HSD-treated group in male and female mice (*N* = 3 individual mice per group). ^*^P < 0.05, ^*##*^*P* < 0.01, ^&&^*P* < 0.01. **(B)** Ratios of heart weight to body weight (HW/BW) of the control group and the HSD-treated group in male and female mice (*N* = 3 individual mice per group). ^*^P < 0.05, ^*##*^*P* < 0.01, ^&&^*P* < 0.01. **(C–E)** Quantification of echocardiographic parameters of the control group and the HSD-treated group in male and female mice (*N* = 3 individual mice per group). ^*^P < 0.05, ^*##*^*P* < 0.01, ^&&^*P* < 0.01, ^*###*^*P* < 0.001, ^&*&&*^*P* < 0.001. **(G)** Representative echocardiography of the control group and the HSD-treated group in male and female mice. **(F)** Cell size of the control group and the HSD-treated group in male and female mice (*N* = 3 individual mice per group). ns, not statistically significant, ^*^P < 0.05, ^&^*P* < 0.05, ^*###*^*P* < 0.001. **(H)** Representative micrographs of cardiac sections stained with HE staining in male and female mice. The scale bar represents 20 μm.

To investigate whether HSD affects the weight and eating habits of mice and any sex-specific differences, the body weight of mice after 8 weeks of administering an HSD was quantified. A two-way ANOVA with two between-subjects factors (sex with two levels, and HSD with two levels, non-HSD treated and HSD-treated) demonstrated a significant sex × HSD interaction (*P* = 0.04) as well as a significant main effect of sex (*P* < 0.001) and HSD (*P* = 0.004) on body weight. These analyses revealed that the body weight of HSD-treated mice was significantly lower than that of the normal group in both male and female mice (Figure 2A), in accordance with a previous report (34). To investigate the effect of HSD on the heart, the hearts of male and female mice were weighed, and the ratios of heart weight to body weight (HW/BW) were calculated. A two-way ANOVA demonstrated a significant main effect of HSD (*P* = 0.002) and sex (*P* = 0.024), but not sex × HSD interaction (*P* = 0.067) on ratios of HW/BW (Figure 2B). To further investigate whether HSD affects the cardiac dysfunction, the fractional shortening (FS), left ventricular (LV) internal dimension at end-diastole (LVIDd) and diastolic left ventricular thickness (LVPWd) were collected. Compared to the normal diet group, the HSD-treated group showed significant decreases in FS and significantly altered both in male and female mice (Figures 2C-E,G). A two-way ANOVA demonstrated (1) a significant main effect of HSD (*P* < 0.001) and sex (*P* = 0.031), but not sex × HSD interaction (*P* = 0.082) on FS; (2) a significant main effect of HSD (*P* = 0.003) and sex (*P* = 0.034), but not sex × HSD interaction (*P* = 0.067) on LVIDd; (3) a significant sex × HSD interaction (*P* = 0.012) as well as a significant main effect of sex (*P* = 0.044) and HSD (*P* = 0.006) on LVPWd. To study the effect of HSD on cardiomyocyte size, HE staining was performed. A two-way ANOVA demonstrated a significant sex × HSD interaction (*P* = 0.037) as well as a significant main effect of HSD (*P* < 0.001) on cell size. In males, the cell size of cardiomyocytes (Figures 2F,H) in the HSD-treated group was significantly increased compared to the normal group. There was also a significant difference in the size of cardiomyocytes in HSD-treated female mice compared to the normal group. We also found that the cardiomyocyte size of male mice was slightly greater than that of female mice in the normal group. Collectively, these results demonstrated that excessive sodium alters the structure and function of the heart with sex-specific changes.

### Excessive Sodium Intake Has a Sex-Specific Effect on the Transcriptome of the Mouse Heart

**Figure 3:**
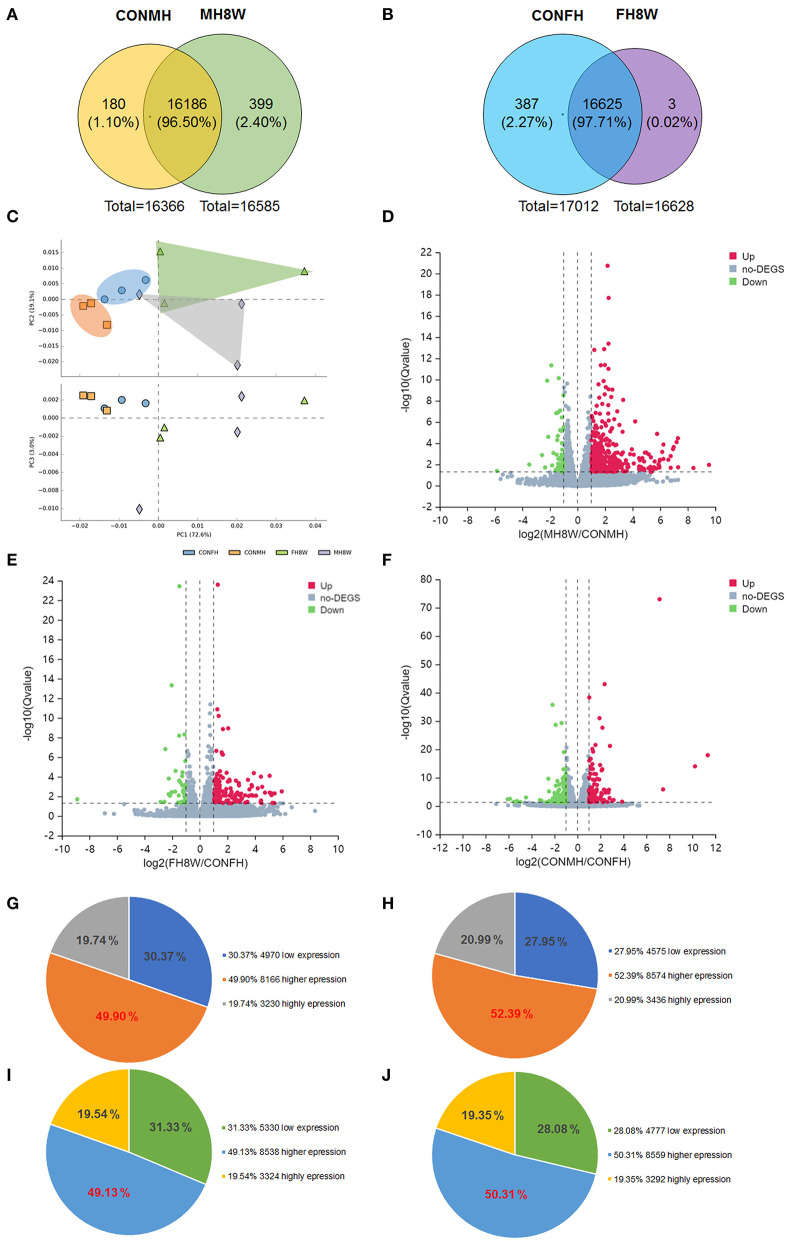
Excessive sodium intake alters the transcriptome composition of mouse heart. **(A)** Venn diagram of the transcriptome for normal and HSD-treated heart in male mice (*N* = 3 individual mice per group). **(B)** Venn diagram of the transcriptome for normal and HSD-treated heart in female mice (*N* = 3 individual mice per group). **(C)** Principal component analysis (PCA) scatterplot between PC1, PC2, and PC3 for total gene expression of the heart in normal male mice, HSD-treated male mice, normal female mice, and HSD-treated female mice, respectively. Blue dots represent the control group of male mice, green dots represent the HSD-treated male mice, orange dots represent the control group of female mice, and lavender dots represent the control group; *N* = 3 individual mice per group. **(D)** Volcano plot of RNA-seq data for heart transcripts in normal male mice and HSD-treated male mice (*N* = 3 individual mice per group). The X-axis represents fold changes. Red and blue dots represent up-regulated genes and green dots represent down-regulated genes. The Y-axis represents -log 10 (Q) value. **(E)** Volcano plot of RNA-seq data for heart transcripts in normal female mice and high salt-treated female mice (*N* = 3 individual mice per group). The X-axis represents fold change. Red and blue dots represent up-regulated genes and green dots represent down-regulated genes. The Y-axis represents the -log 10 (Q) value. **(F)** Volcano plot of RNA-seq data for heart transcripts in normal male mice and normal female mice (*N* = 3 individual mice per group). The X-axis represents fold change. Red and blue dots represent up-regulated genes and green dots represent down-regulated genes. The Y-axis represents the -log_10_ (Q) value. **(G–J)** Pie charts of transcriptome composition in normal male mice **(G)**, HSD-treated male mice **(H)**, normal female mice **(I)**, and HSD-treated female mice **(J)**.

To investigate the effect of excessive sodium intake on the transcriptome, changes in gene expression of male and female mice fed with an HSD were delineated. For male mice, 16,366 and 16,585 gene transcripts were detected in the hearts of the control (CONMH) and high-salt-treated (MH8W) groups, respectively (Figure 3A); for female mice, 17,012 and 16,628 gene transcripts were detected in the heart of the control (CONFH) group and high-salt-treated (FH8W) group, respectively (Figure 3B). After HSD treatment in male mice, the Venn diagram shows that 180 (1.10%) genes were exclusively included in the CONMH group, 16,186 (95.50%) genes were common to both CONMH and MH8W groups, and 399 (2.40%) genes were exclusively included in the MH8W group (Figure 3A). After HSD treatment in female mice, the Venn diagram shows that 387 (2.27%) genes were exclusively included in the CONFH group, 16,625 (97.71%) common genes were included in the CONFH and FH8W groups, and 3 (0.02%) genes were exclusively included in the FH8W group (Figure 3B).

Principal component analysis was used for data visualization to determine transcriptomic differences between the CONMH and MH8W groups, and CONFH and FH8W groups, respectively (35). The dimensional reduction was performed on 12 variables. As shown in Figure 3C, the 12 variables in the three principal components (PC1, PC2, and PC3) accounted for 72.60, 19.10, and 3.00% of the total variance, respectively, which shows that the four study groups are clustered within and separated between groups. The PCA results revealed that excessive sodium intake alters the heart transcriptome and that the heart transcriptome is different between male and female mice. To further explore the effect of excessive sodium intake on cardiac transcriptome composition, a volcano plot of DEGs (*Q* value <0.05) was compared between the CONMH and MH8W groups, CONFH and FH8W groups, and CONMH and CONFH groups, respectively. As shown in Figure 3D, 534 DEGs were identified, which included 485 upregulated and 49 downregulated genes between the CONMH and MH8W groups (Supplementary Table S2). As shown in Figure 3E, 155 DEGs were identified, including 117 upregulated and 38 downregulated genes between the CONFH and FH8W groups (Supplementary Table S2). As shown in Figure 3F, 238 DEGs were identified, including 99 upregulated and 139 downregulated genes between the CONMH and CONFH groups (Supplementary Table S3). To further explore the effect of excessive sodium intake on the cardiac transcriptome, we analyzed gene expression levels in the CONMH, MH8W, CONFH, and FH8W groups. The transcripts were classified into three categories according to their respective groups: low expression genes (0 < FPKM < 1), higher expression genes (1 ≤ FPKM < 20), and high expression genes (FPKM ≥ 20), in accordance with previous reports (36, 37). For male mice, of the 16,366 transcripts in the CONMH group, low expression genes, higher expression genes, and high expression genes accounted for 30.37% (4,970), 49.90% (8,166), and 19.74% (3,230), respectively (Figure 3G). Of the 16,585 transcripts in the MH8W group, low expression genes, higher expression genes, and high expression genes accounted for 27.95% (4,575), 52.39% (8,574), and 20.99% (3,436), respectively (Figure 3H). However, for the female mice, of the 17,012 transcripts in the CONFH group, low expression genes, higher expression genes, and high expression genes accounted for 31.33% (5,330), 49.13% (8,358), and 19.54% (3,324), respectively (Figure 3I), and of the 16,628 transcripts in the FH8W group, low expression genes, higher expression genes, and high expression genes accounted for 28.08% (4,777), 50.31% (8,559), and 19.35% (3,292), respectively (Figure 3J). Collectively, these results demonstrated that excessive sodium feeding altered the composition of the cardiac transcriptome with sex-specific changes.

### Excessive Sodium Intake Alters the KEGG Pathway of Mouse Heart

**Figure 4:**
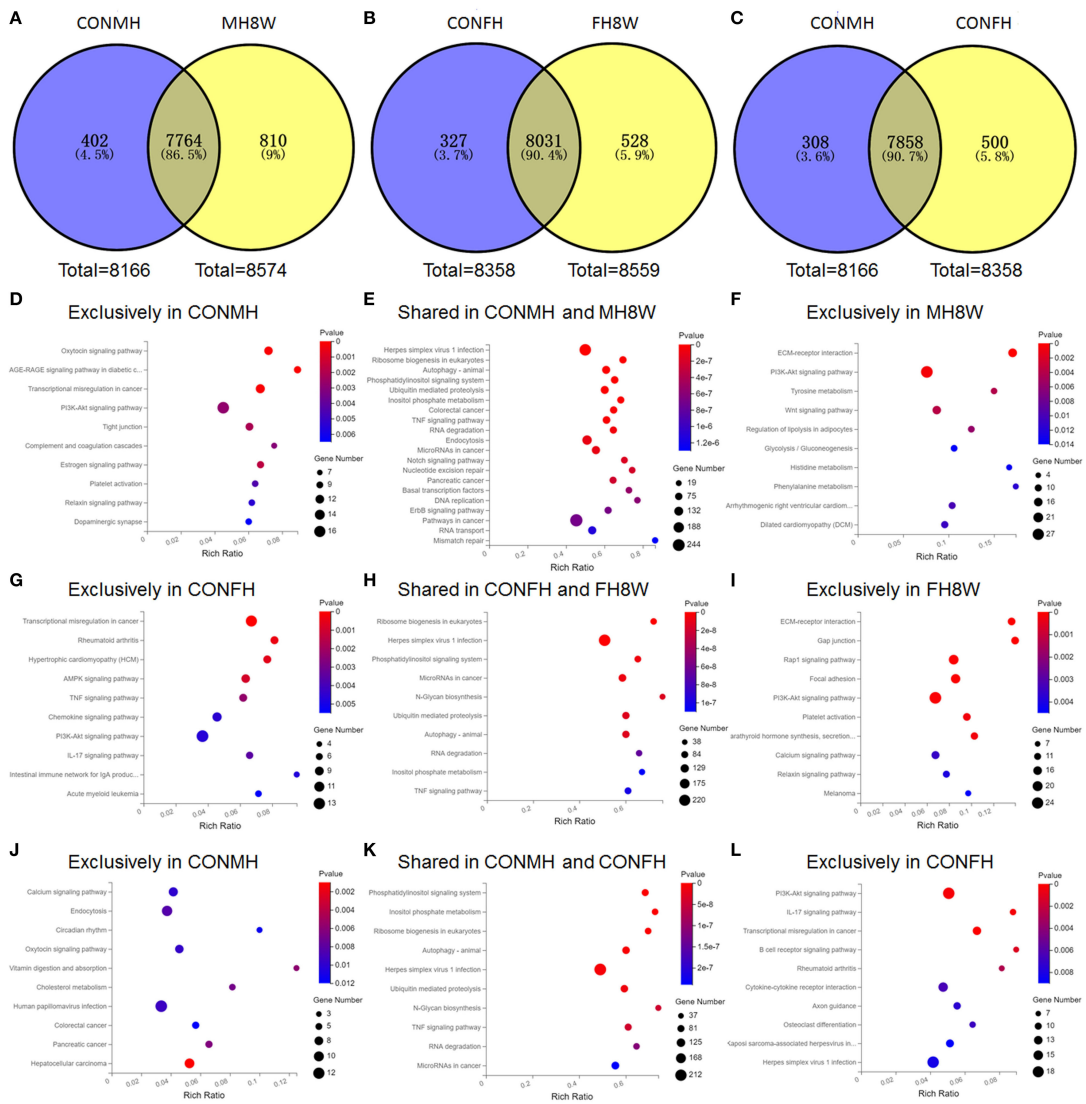
Excessive sodium intake alters KEGG pathways with sex-specific effects in mouse heart. **(A)** Venn diagram of higher expression genes for normal and HSD-treated heart tissue in male mice (*N* = 3 individual mice per group). **(B)** Venn diagram of higher expression genes for normal and HSD-treated heart tissue in female mice (*N* = 3 individual mice per group). **(C)** Venn diagram of higher expression genes for normal heart tissue of male and female mice (*N* = 3 individual mice per group). **(D)** Gene annotation of top 10 KEGG pathways enriched in higher expression genes unique to the normal heart tissue of male mice with *P* < 0.05 (*N* = 3 individual mice per group). **(E)** Gene annotation of top 10 KEGG pathways enriched in higher expression genes shared in the normal and HSD-treated heart tissue in male mice with *P* < 0.05 (*N* = 3 individual mice per group). **(F)** Gene annotation of top 10 KEGG pathways enriched in higher expression genes unique to the HSD-treated heart tissue in male mice with *P* < 0.05 (*N* = 3 individual mice per group). **(G)** Gene annotation of top 10 KEGG pathways enriched in higher expression genes unique to the normal heart tissue of female mice with *P* < 0.01 (*N* = 3 individual mice per group). **(H)** Gene annotation of top 10 KEGG pathways enriched in higher expression genes shared in the normal and HSD-treated heart tissue in female mice with *P* < 0.05 (*N* = 3 individual mice per group). **(I)** Gene annotation of top 10 KEGG pathways enriched in higher expression genes unique to the HSD-treated heart tissue in female mice with *P* < 0.05 (*N* = 3 individual mice per group). **(J)** Gene annotation of top 10 KEGG pathways enriched in higher expression genes unique to the normal heart tissue of male mice with *P* < 0.05 (*N* = 3 individual mice per group). **(K)** Gene annotation of top 10 KEGG pathways enriched in higher expression genes shared in the normal heart tissue of male mice and normal heart tissue of female mice with *P* < 0.05 (*N* = 3 individual mice per group). **(L)** Gene annotation of top 10 KEGG pathways enriched in higher expression genes unique to the normal heart tissue of female mice with *P* < 0.05 (*N* = 3 individual mice per group).

To investigate the effect of excessive sodium intake on the higher expression genes, we compared the difference between the CONMH and MH8W groups, and CONFH and FH8W groups using Venn plots. As shown in Figure 4A, the Venn diagram shows that 402 (4.50%) genes were exclusively included in the CONMH group 7,764 (86.50%) genes were common to the CONMH and MH8W groups, and 810 (9.00%) genes were exclusively included in the MH8W group. However, after HSD treatment in female mice, the Venn diagram shows that 327 (3.70%) genes were exclusively included in the CONFH group 8,031 (90.40%) genes were common to the CONFH and FH8W groups, and 528 (5.90%) genes were exclusively included in the MH8W group (Figure 4B). We further compared the differences in genes with higher expression in male and female mice. As shown in Figure 4C, the Venn diagram shows that 308 (3.60%) genes were exclusively included in the CONMH group 7,858 (90.70%) genes were common to the CONMH and CONFH groups, and 500 (5.80%) genes were exclusively included in the CONFH group.

To evaluate the effect of excessive sodium intake on the KEGG pathways of the genes with higher expression in the heart, we carried out KEGG enrichment analyses on the genes unique to the CONMH group, unique to the MH8W group, unique to the CONFH group, unique to the FH8W group, shared between the CONMH and MH8W groups, the CONFH and FH8W groups, and the CONMH and CONFH groups. For male mice, 23 significantly enriched functional pathways (*P* < 0.05) were identified as being unique to the CONMH group and were divided into four categories: (1) cellular processes: tight junction (*P* = 2.27E-03), focal adhesion (*P* = 8.22E-03), and lysosome (*P* = 4.15E-02); (2) environmental information processing: PI3K-akt signaling pathway (*P* = 2.89E-03), ECM-receptor interaction (*P* = 9.86E-03), and Rap1 signaling pathway (*P* = 1.37E-02); (3) human diseases: AGE-RAGE signaling pathway in diabetic complications (*P* = 2.39E-04), transcriptional mis-regulation in cancer (*P* = 3.41E-04), human T-cell leukemia virus 1 infection (*P* = 3.09E-02), and systemic lupus erythematosus (*P* = 2.85E-02); (4) organismal systems: oxytocin signaling pathway (*P* = 3.32E-04), complement and coagulation cascades (*P* = 3.15E-03), estrogen signaling pathway (*P* = 1.86E-03), platelet activation (*P* = 4.45E-03), relaxing signaling pathway (*P* = 5.37E-03), dopaminergic synapse (*P* = 6.44E-03), protein digestion and absorption (*P* = 1.34E-02), cholesterol metabolism (*P* = 1.85E-02), glucagon signaling pathway (*P* = 2.11E-02), circadian rhythm (*P* = 2.38E-02), glutamatergic synapse (*P* = 3.02E-02), cholinergic synapse (*P* = 2.90E-02), and adrenergic signaling in cardiomyocytes (*P* = 3.91E-02) (Figure 4D). A total of 11 significantly enriched pathways (*P* < 0.05) were identified in the shared CONMH and MH8W groups and were divided into five categories: (1) cellular processes: autophagy-animal (*P* = 8.05E-10) and endocytosis (*P* = 1.02E-07); (2) environmental information processing: phosphatidylinositol signaling system (*P* = 1.46E-09) and TNF signaling pathway (*P* = 2.18E-08); (3) genetic information processing: ribosome biogenesis in eukaryotes (*P* = 5.72E-10), ubiquitin mediated proteolysis (*P* = 2.31E-09), and RNA degradation (*P* = 6.35E-08); (4) human diseases: herpes simplex virus 1 infection (*P* = 1.44E-10) and colorectal cancer (*P* = 1.80E-08); (5) metabolism: inositol phosphate metabolism (*P* = 8.35E-09) (Figure 4E). While 11 significantly enriched functional pathways (*P* < 0.05) were identified as being unique to the MH8W group and were divided into four categories: (1) environmental information processing: ECM-receptor interaction (*P* = 1.09E-06), PI3K-Akt signaling pathway (*P* = 5.40E-04), and WNT signaling pathway (*P* = 3.63E-03); (2) human diseases: arrhythmogenic right ventricular cardiomyopathy (ARVC; *P* = 9.27E-03) and dilated cardiomyopathy (DCM; *P* = 1.00E-02); (3) metabolism: tyrosine metabolism (*P* = 3.96E-03), phenylalanine metabolism (*P* = 1.07E-02), histidine metabolism (*P* = 1.25E-02), and glycolysis/gluconeogenesis (*P* = 1.31E-02); (4) organismal systems: regulation of lipolysis in adipocytes (*P* = 5.40E-03) (Figure 4F).

For female mice, 11 significantly enriched functional pathways (*P* < 0.01) were identified as being unique to the CONFH group and were divided into three categories: (1) environmental information processing: AMPK signaling pathway (*P* = 8.81E-04), TNF signaling pathway (*P* = 2.13E-03), PI3K-Akt signaling pathway (*P* = 4.27E-03), and cytokine-cytokine receptor interaction (*P* = 7.40E-03); (2) human diseases: transcriptional mis-regulation in cancer (*P* = 2.66E-05), rheumatoid arthritis (*P* = 4.23E-04), hypertrophic cardiomyopathy (HCM; *P* = 5.97E-04), acute myeloid leukemia (*P* = 5.06E-03), measles (*P* = 7.51E-03), and systemic lupus erythematosus (*P* = 7.51E-03); (3) organismal systems: IL-17 signaling pathway (*P* = 3.24E-03), chemokine signaling pathway (*P* = 4.18E-03), intestinal immune network for IgA production (*P* = 4.33E-03), and adipocytokine signaling pathway (*P* = 5.38E-03) (Figure 4G). A total of 69 significantly enriched functional pathways (*P* < 0.001) were identified in the shared CONFH and FH8W groups and were divided into six categories: (1) cellular processes: autophagy-animal (*P* = 1.33E-08), endocytosis (*P* = 1.63E-05), cell cycle (*P* = 1.69E-05), adherens junction (*P* = 8.86E-05), lysosome (*P* = 1.17E-04), apoptosis (*P* = 1.34E-04), signaling pathways regulating pluripotency of stem cells (*P* = 6.37E-04), and apoptosis-fly (*P* = 9.50E-04); (2) environmental information processing: phosphatidylinositol signaling system (*P* = 6.48E-08), TNF signaling pathway (*P* = 1.84E-07), notch signaling pathway (*P* = 2.33E-07), MAPK signaling pathway (*P* = 4.83E-07), ERBB signaling pathway (*P* = 5.01E-07), WNT signaling pathway (*P* = 2.14E-06), hippo signaling pathway-fly (*P* = 4.95E-05), RAS signaling pathway (*P* = 5.79E-05), rap1 signaling pathway (*P* = 7.87E-05), mTOR signaling pathway (*P* = 1.29E-04), NF-kappa B signaling pathway (*P* = 7.18E-04), and hippo signaling pathway-multiple species (*P* = 3.40E-10); (3) genetic information processing: ribosome biogenesis in eukaryotes (*P* = 6.93E-05), ubiquitin mediated proteolysis (*P* = 2.04E-04), RNA degradation (*P* = 2.59E-04), DNA replication (*P* = 3.89E-04), basal transcription factors (*P* = 4.25E-04), nucleotide excision repair (*P* = 4.46E-04), RNA transport (*P* = 9.61E-04), mismatch repair (*P* = 2.88E-06), non-homologous end-joining (*P* = 1.32E-05), base excision repair (*P* = 9.84E-05), spliceosome (*P* = 4.92E-04), Fanconi anemia pathway (*P* = 6.30E-04), and aminoacyl-tRNA biosynthesis (*P* = 8.04E-04); (4) human diseases: herpes simplex virus 1 infection (*P* = 3.40E-10), microRNAs in cancer (*P* = 7.55E-09), pathways in cancer (*P* = 1.32E-07), colorectal cancer (*P* = 6.54E-07), pancreatic cancer (*P* = 6.45E-06), hepatitis B (*P* = 1.19E-05), endocrine resistance (*P* = 1.93E-05), breast cancer (*P* = 6.12E-05), chronic myeloid leukemia (*P* = 1.23E-04), small cell lung cancer (*P* = 1.70E-04), prostate cancer (*P* = 1.95E-04), endometrial cancer (*P* = 1.96E-04), hepatocellular carcinoma (*P* = 2.80E-04), insulin resistance (*P* = 3.82E-04), AGE-RAGE signaling pathway in diabetic complications (*P* = 3.82E-04), non-small cell lung cancer (*P* = 4.08E-04), human papillomavirus infection (*P* = 5.64E-04), and choline metabolism in cancer (*P* = 6.17E-04); (5) metabolism: N-Glycan biosynthesis (*P* = 1.49E-08), inositol phosphate metabolism (*P* = 1.07E-07), pyrimidine metabolism (*P* = 2.00E-06), lysine degradation (*P* = 1.27E-05), terpenoid backbone biosynthesis (*P* = 5.54E-05), glycosylphosphatidylinositol (GPI)-anchor biosynthesis (*P* = 6.93E-05), one carbon pool by folate (*P* = 2.04E-04), purine metabolism (*P* = 2.59E-04), amino sugar and nucleotide sugar metabolism (*P* = 3.89E-04), other types of O-glycan biosynthesis (*P* = 4.25E-04), other glycan degradation (*P* = 4.46E-04), and seleno-compound metabolism (*P* = 9.61E-04); (6) organismal systems: axon guidance (*P* = 2.88E-06), FC gamma R-mediated phagocytosis (*P* = 1.32E-05), thyroid hormone signaling pathway (*P* = 9.84E-05), chemokine signaling pathway (*P* = 4.92E-04), B cell receptor signaling pathway (*P* = 6.30E-04), osteoclast differentiation (*P* = 8.04E-04) (Figure 4H). While 20 significantly enriched functional pathways (*P* < 0.01) were identified as being unique to the FH8W group and were divided into five categories: (1) cellular processes: gap junction (*P* = 5.33E-06) and focal adhesion (*P* = 4.93E-05); (2) environmental information processing: ECM-receptor interaction (*P* = 6.80E-06), Rap1 signaling pathway (*P* = 3.88E-05), PI3K-Akt signaling pathway (*P* = 7.46E-05), calcium signaling pathway (*P* = 3.39E-03), MAPK signaling pathway (*P* = 4.49E-03), and WNT signaling pathway (*P* = 6.38E-03); (3) human diseases: melanoma (*P* = 4.34E-03), hypertrophic cardiomyopathy (HCM; *P* = 4.38E-03), dilated cardiomyopathy (DCM; *P* = 5.33E-03), herpes simplex virus 1 infection (*P* = 6.02E-03), and arrhythmogenic right ventricular cardiomyopathy (ARVC; *P* = 6.29E-03); (4) metabolism: tyrosine metabolism (*P* = 5.33E-03); (5) organismal systems: parathyroid hormone synthesis, secretion and action (*P* = 2.29E-04), platelet activation (*P* = 2.29E-04), relaxing signaling pathway (*P* = 3.79E-03), oxytocin signaling pathway (*P* = 4.36E-03), thyroid hormone signaling pathway (*P* = 6.22E-03), and circadian entrainment (*P* = 6.84E-03) (Figure 4I).

These data showed that male and female mice expressed different transcripts; therefore, we further compared KEGG pathways using KEGG enrichment analyses. A total of eight significantly enriched functional pathways (*P* < 0.01) were identified as being unique to the CONMH group and were divided into four categories: (1) cellular processes: endocytosis (*P* = 7.91E-03); (2) environmental information processing: calcium signaling pathway (*P* = 9.34E-03); (3) human diseases: hepatocellular carcinoma (*P* = 1.23E-03), pancreatic cancer (*P* = 6.12E-03), and human papillomavirus infection (*P* = 8.82E-03); (4) organismal systems: vitamin digestion and absorption (*P* = 5.66E-03), cholesterol metabolism (*P* = 6.60E-03), and oxytocin signaling pathway (*P* = 9.12E-03) (Figure 4J). Top 10 KEGG pathways were shown (Figure 4K), which enriched in higher expression genes shared in the normal heart tissue of male mice and normal heart tissue of female mice with *P* < 0.05. A total of 12 significantly enriched functional pathways (*P* < 0.01) were identified as being unique to the CONFH group and were divided into three categories: (1) environmental information processing: PI3K-Akt signaling pathway (*P* = 8.93E-04), cytokine-cytokine receptor interaction (*P* = 5.96E-03), and WNT signaling pathway (*P* = 9.59E-03); (2) human diseases: transcriptional mis-regulation in cancer (*P* = 6.20E-04), rheumatoid arthritis (*P* = 2.92E-03), herpes simplex virus 1 infection (*P* = 7.40E-03), Kaposi sarcoma-associated herpesvirus infection (*P* = 8.08E-03), and microRNAs in cancer (*P* = 9.96E-03); (3) organismal systems: IL-17 signaling pathway (*P* = 8.99E-04), B cell receptor signaling pathway (*P* = 1.67E-03), osteoclast differentiation (*P* = 6.27E-03), and axon guidance (*P* = 6.74E-03) (Figure 4L). Altogether, these results revealed that excessive sodium intake alters KEGG pathways of the mouse heart with sex-specific changes.

### Excessive Sodium Intake Induces Cardiovascular Disease in Mouse

**Figure 5:**
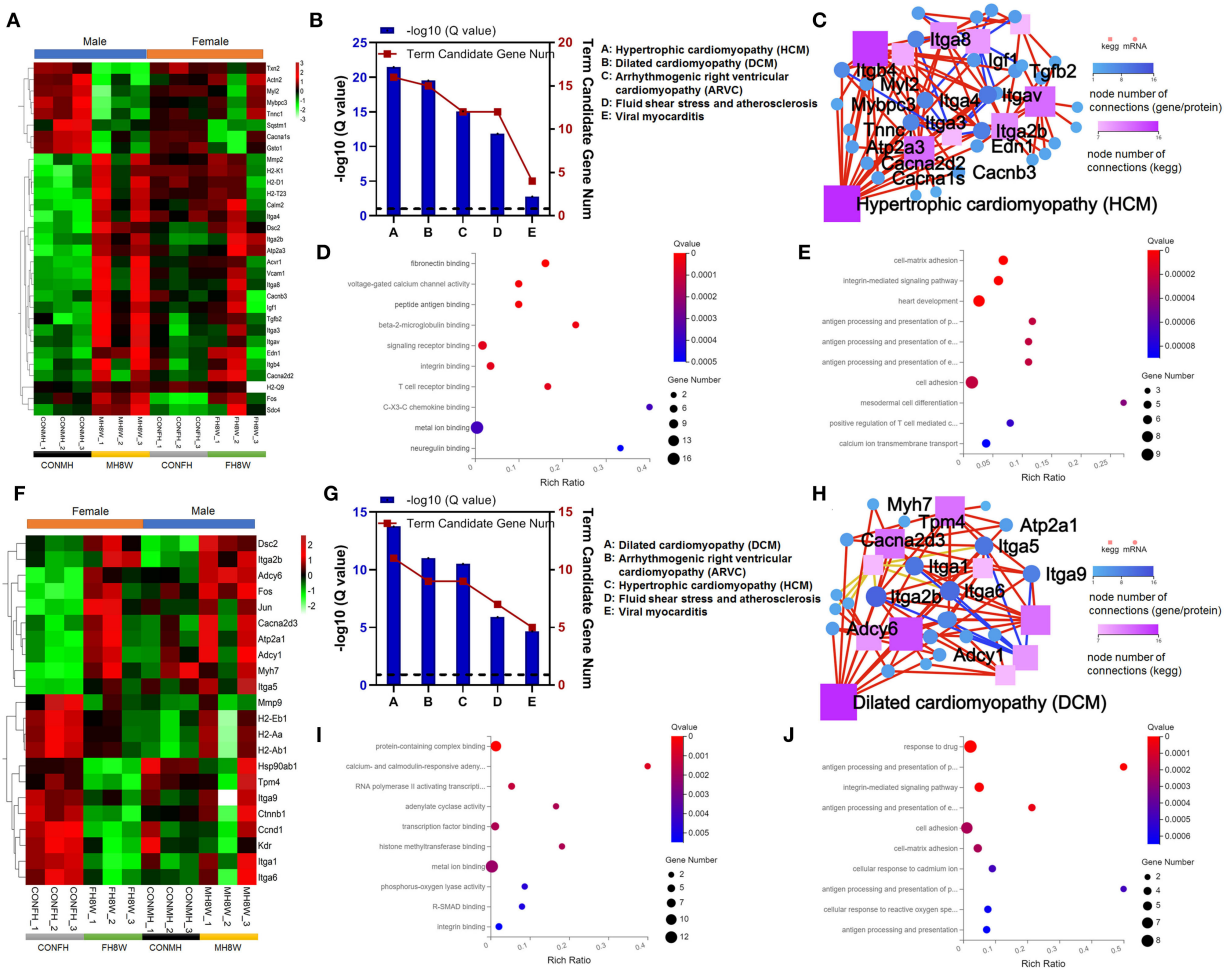
Excessive sodium intake alters the expression profile of cardiovascular disease-associated genes in the mouse heart. **(A)** Heatmaps of the cardiovascular disease-associated genes in heart tissue of male mice and the same genes were also present in female mice (*N* = 3 individual mice per group). The color bar indicates the scale used to show the expression of genes with the expression range normalized to ± 3. **(B)** Gene annotation of KEGG pathways enriched in cardiovascular disease-associated genes for heart tissue of male mice, with *Q* < 0.05 (*N* = 3 individual mice per group). **(C)** The KEGG pathway relationship network corresponds to the KEGG pathway of the selected gene in cardiovascular disease-associated genes for heart tissue of male mice (*N* = 3 individual mice per group). The KEGG pathway relationship network was sorted by the number of genes on the pathway and displayed the 10 pathways with the largest number of genes. **(D)** Gene annotation of the molecular function of GO enriched in cardiovascular disease-associated genes for heart tissue of male mice, with *Q* < 0.001 (*N* = 3 individual mice per group). The top 10 terms are shown. **(E)** Gene annotation of biological processes of GO enriched in cardiovascular disease-associated genes for heart tissue of male mice, with *Q* < 0.001 (*N* = 3 individual mice per group). The top 10 terms are shown. **(F)** Heatmaps of the cardiovascular disease-associated genes in heart tissue of female mice and the same genes were also present in male mice (*N* = 3 individual mice per group). The color bar indicates the scale used to show the expression of genes with the expression range normalized to ± 2. **(G)** Gene annotation of KEGG pathways enriched in cardiovascular disease-associated genes for heart tissue of female mice, with *Q* < 0.05 (*N* = 3 individual mice per group). **(H)** The KEGG pathway relationship network corresponds to the selected gene of cardiovascular disease-associated genes in the heart tissue of female mice (*N* = 3 individual mice per group). The KEGG pathway relationship network was sorted by the number of genes on the pathway and the 10 pathways with the largest number of genes are displayed. **(I)** Gene annotation of the molecular function of GO enriched terms for cardiovascular disease-associated genes in heart tissue of female mice, with *Q* < 0.01 (*N* = 3 individual mice per group). The top 10 terms were shown. **(J)** Gene annotation of a biological process of GO enriched terms for cardiovascular disease-associated genes for heart tissue of female mice, with *Q* < 0.001 (*N* = 3 individual mice per group).

Evidence from clinical trials indicates that excessive sodium intake is associated with cardiovascular disease (38, 39). To determine the effect of excessive sodium feeding, we first compared the expression of cardiovascular disease-related transcripts between normal and high-salt-treated hearts in male mice. As shown in Figure 5A (left), in male mice, cardiovascular disease-related transcripts were significantly different levels of expression in high-salt-treated compared with normal hearts. However, we also observed that the same transcripts of cardiovascular disease were slightly different levels of expression in high-salt-treated hearts compared with that in normal hearts in female mice (Figure 5A, right). The KEGG functional enrichment analysis of these transcripts showed that the cardiovascular disease-related pathways mainly included hypertrophic cardiomyopathy (HCM; *Q* = 3.17E-22), dilated cardiomyopathy (DCM; *Q* = 2.94E-20), arrhythmogenic right ventricular cardiomyopathy (ARVC; *Q* = 8.09E-16), fluid shear stress and atherosclerosis (*Q* = 1.37E-12), and viral myocarditis (*Q* = 1.65E-03) (Figure 5B). The KEGG pathway relationship network was then visualized and plotted. As shown in Figure 5C by the node number of connections, HCM is the most common KEGG pathway. To further investigate the molecular function and biological process of cardiovascular disease-related transcripts between normal and high-salt-treated hearts in male mice, GO enrichment analysis was performed. As shown in Figure 5D, molecular function can be divided into two categories: (1) binding, fibronectin binding (*Q* = 1.43E-07), peptide antigen binding (*Q* = 1.91E-05), beta-2-microglobulin binding (*Q* = 3.35E-05), signaling receptor binding (*Q* = 5.43E-05), integrin binding (*Q* = 5.43E-05), T cell receptor binding (*Q* = 5.43E-05), CX3C chemokine binding (*Q* = 3.67E-04), metal ion binding (*Q* = 3.67E-04), neuregulin binding (*Q* = 4.95E-04), protein-containing complex binding (*Q* = 6.58E-04), and TAP binding (*Q* = 9.87E-04); (2) transporter activity, voltage-gated calcium channel activity (*Q* = 1.91E-05). As shown in Figure 5E, the biological process can be divided six categories: (1) biological regulation: cell-matrix adhesion (*Q* = 2.31E-06), cell adhesion (*Q* = 2.33E-05), heterotypic cell-cell adhesion (*Q* = 3.57E-04), endocardial cushion fusion (*Q* = 4.71E-04), integrin-mediated signaling pathway (*Q* = 2.31E-06), positive regulation of T cell mediated cytotoxicity (*Q* = 7.00E-05), positive regulation of cell migration (*Q* = 1.10E-04), and positive regulation of epithelial to mesenchymal transition involved in endocardial cushion formation (*Q* = 6.21E-04); (2) cellular process: mesodermal cell differentiation (*Q* = 4.31E-05), cell-cell adhesion in response to extracellular stimulus (*Q* = 1.15E-04), endodermal cell differentiation (*Q* = 3.76E-04), positive regulation of vascular smooth muscle cell proliferation (*Q* = 6.21E-04), and cellular response to amyloid-beta (*Q* = 7.42E-04); (3) developmental process: heart development (*Q* = 2.31E-06), inner ear development (*Q* = 2.44E-04), ventricular cardiac muscle tissue morphogenesis (*Q* = 4.41E-04), and atrial septum primum morphogenesis (*Q* = 6.21E-04); (4) immune system process: antigen processing and presentation of peptide antigen *via* MHC class I (*Q* = 2.33E-05), antigen processing and presentation of endogenous peptide antigen *via* MHC class Ib (*Q* = 2.33E-05), antigen processing and presentation of endogenous peptide antigen *via* MHC class I *via* ER pathway, TAP-independent (*Q* = 2.33E-05), and antigen processing and presentation of endogenous peptide antigen *via* MHC class I *via* ER pathway, TAP-dependent (*Q* = 6.21E-04); (5) localization: calcium ion transmembrane transport (*Q* = 8.76E-05) and cell migration (*Q* = 1.15E-04); (6) response to stimulus: response to drug (*Q* = 4.71E-04) and response to hypoxia (*Q* = 4.81E-04).

To further elucidate the effect of excessive sodium intake, we next compared the expression of cardiovascular disease-related transcripts between normal and high-salt-treated hearts in female mice. As shown in Figure 5F (left), in female mice cardiovascular disease-related transcripts were significantly different in high-salt-treated hearts compared with normal hearts. However, we also observed that the same transcripts of cardiovascular disease were slightly different in high-salt-treated hearts compared with normal hearts in male mice (Figure 5F, right). KEGG functional enrichment analysis of these transcripts showed that the cardiovascular disease-related pathways mainly included: DCM (*Q* = 1.77E-14), ARVC (*Q* = 9.55E-12), HCM (*Q* = 3.03E-11), fluid shear stress and atherosclerosis (*Q* = 1.25E-06), and viral myocarditis (*Q* = 2.08E-05) (Figure 5G). KEGG analysis also showed that the pathway with the highest number of nodes was DCM (Figure 5H). GO analysis was used to compare molecular function and biological process of cardiovascular disease-related transcripts between normal hearts and high-salt-treated hearts in female mice. As shown in Figure 5I, the molecular function can be divided two categories: (1) binding: protein-containing complex binding (HSD-treated female mice vs. HSD-treated male mice, *Q* value 1.59E-06 vs. 6.58E-04), RNA polymerase II activating transcription factor binding (*Q* = 1.20E-03), transcription factor binding (*Q* = 1.63E-03), histone methyltransferase binding (*Q* = 1.63E-03), metal ion binding (*Q* value 2.01E-03 vs. 3.67E-04), R-SMAD binding (*Q* = 4.48E-03), integrin binding (*Q* value 5.07E-03 vs. 5.43E-05), laminin binding (*Q* = 5.07E-03), disordered domain-specific binding (*Q* = 7.41E-03), peptide antigen binding (*Q* value 7.99E-03 vs. 1.91E-05), and protein binding (*Q* = 8.44E-03); (2) transporter activity: calcium- and calmodulin-responsive adenylate cyclase activity (*Q* = 6.81E-04), adenylate cyclase activity (*Q* = 1.63E-03), and phosphorus-oxygen lyase activity (*Q* = 4.26E-03). As shown in Figure 5J, the biological process can be divided four categories: (1) biological regulation: cell adhesion (HSD-treated female mice vs. HSD-treated male mice, *Q* value 2.03E-04 vs. 2.33E-05), cell-matrix adhesion (*Q* value 2.03E-04 vs. 2.31E-06), and integrin-mediated signaling pathway (*Q* value 1.10E-05 vs. 2.31E-06); (2) cellular process: cellular response to cadmium ion (*Q* = 4.43E-04) and cellular response to reactive oxygen species (*Q* = 5.75E-04); (3) immune system process: antigen processing and presentation of peptide or polysaccharide antigen *via* MHC class II (*Q* = 5.79E-06), antigen processing and presentation of exogenous peptide antigen *via* MHC class II (*Q* = 5.24E-05), antigen processing and presentation of peptide antigen (*Q* = 4.47E-04), and antigen processing and presentation (*Q* = 6.03E-04); (4) response to stimulus: response to drug (*Q* value 9.87E-07 vs. 4.71E-04). Collectively, these results demonstrate that excessive sodium intake can easily induce cardiovascular disease.

### Excessive Sodium Intake Alters the Expression Profile of Fibrosis in Mouse Heart

**Figure 6:**
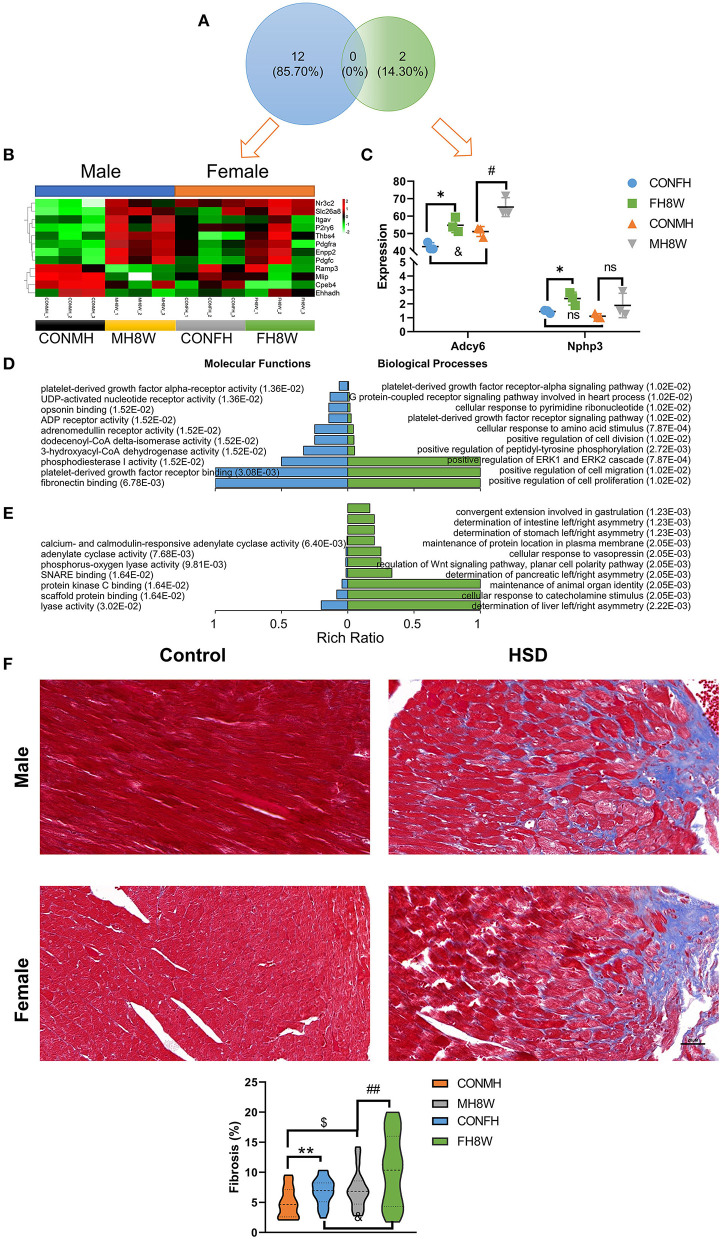
Excessive sodium intake alters the expression profile of fibrosis-associated genes in mouse heart. **(A)** Venn diagram of significantly different genes related to fibrosis for HSD-treated heart tissue in male mice and female mice compared to the normal heart tissue in male and female mice (*N* = 3 individual mice per group). **(B)** Heatmaps of the fibrosis-associated genes in heart tissue of male mice and the same genes were also present in female mice (*N* = 3 individual mice per group). The color bar indicates the scale used to show the expression of genes with the expression range normalized to ± 2. **(C)** The expression of significantly different genes related to fibrosis for HSD-treated female male mice compared to the normal heart tissue in female and male mice (*N* = 3 individual mice per group). ns, not statistically significant, *P < 0.05, ^#^*P* < 0.05, ^&^*P* < 0.05. **(D)** Gene annotation of molecular function and biological process of GO enriched terms for fibrosis-associated genes for heart tissue of male mice, with *Q* < 0.01 (*N* = 3 individual mice per group). The top 10 terms were shown. **(E)** Gene annotation of molecular function (*Q* < 0.05) and biological process (*Q* < 0.01) of GO enriched terms for fibrosis-associated genes for the heart of male mice (*N* = 3 individual mice per group). The top 10 terms were shown. **(F)** Representative immunohistochemical images of normal heart and HSD-treated heart in male and female mice (*N* = 3 individual mice per group). The scale bar represents 20 μm. Quantification of fibrosis measured as a percentage of total myocardial area in heart of the control group compared with the HSD-treated group. ^$^*P* < 0.05, ^&^*P* < 0.05, **P < 0.01, ^##^*P* < 0.01.

As previously reported, HSDs have been closely linked to the occurrence and development of cardiac fibrosis in normotensive and hypertensive rats (40, 41). To verify the effect of excessive sodium feeding on the fibrosis-associated molecular signature in the hearts of male and female mice, we investigated the changes in fibrosis-associated transcript levels in the heart. For male mice, we found that the expression of 12 fibrosis-associated transcripts was significantly different; however, only two fibrosis-associated transcripts were significantly different in female mice (*Q* < 0.05) (Figure 6A). As shown in Figures 6B,C independent of sex, the expression of fibrosis-related genes in HSD mice is higher than that in the normal group, and there is a disorder. To further study the molecular function and biological processes of these fibrosis-related genes, we performed a GO enrichment analysis. The molecular functions and biological processes of these genes were affected by HSD treatment in male mice (Figure 6D and Supplementary Tables S4, S5) and female mice (Figure 6E and Supplementary Table S6).

To further investigate whether excessive sodium intake accelerates the formation of fibrosis in the heart, we used IHC to show the fibrotic level of the heart in male and female mice. A two-way ANOVA demonstrated a significant main effect of sex (*F* = 9.958; *P* = 0.002) as well as a significant main effect of HSD (*F* = 12.523; *P* = 0.001), but not sex × HSD interaction (*F* = 0.941; *P* = 0.335). As shown in Figure 6F, HSD treatment significantly increased cardiac fibrosis in both male and female mice (*P* < 0.05). Further studies have found that fibrotic changes caused by HSD in the heart of female mice were approximately twice that of male mice (1.75 vs. 2.74%) (Figure 6F). Collectively, these results demonstrate that excessive sodium intake can easily induce cardiac fibrosis.

### Excessive Sodium Intake Alters the Expression Profile of Metabolism in Mouse Heart

**Figure 7:**
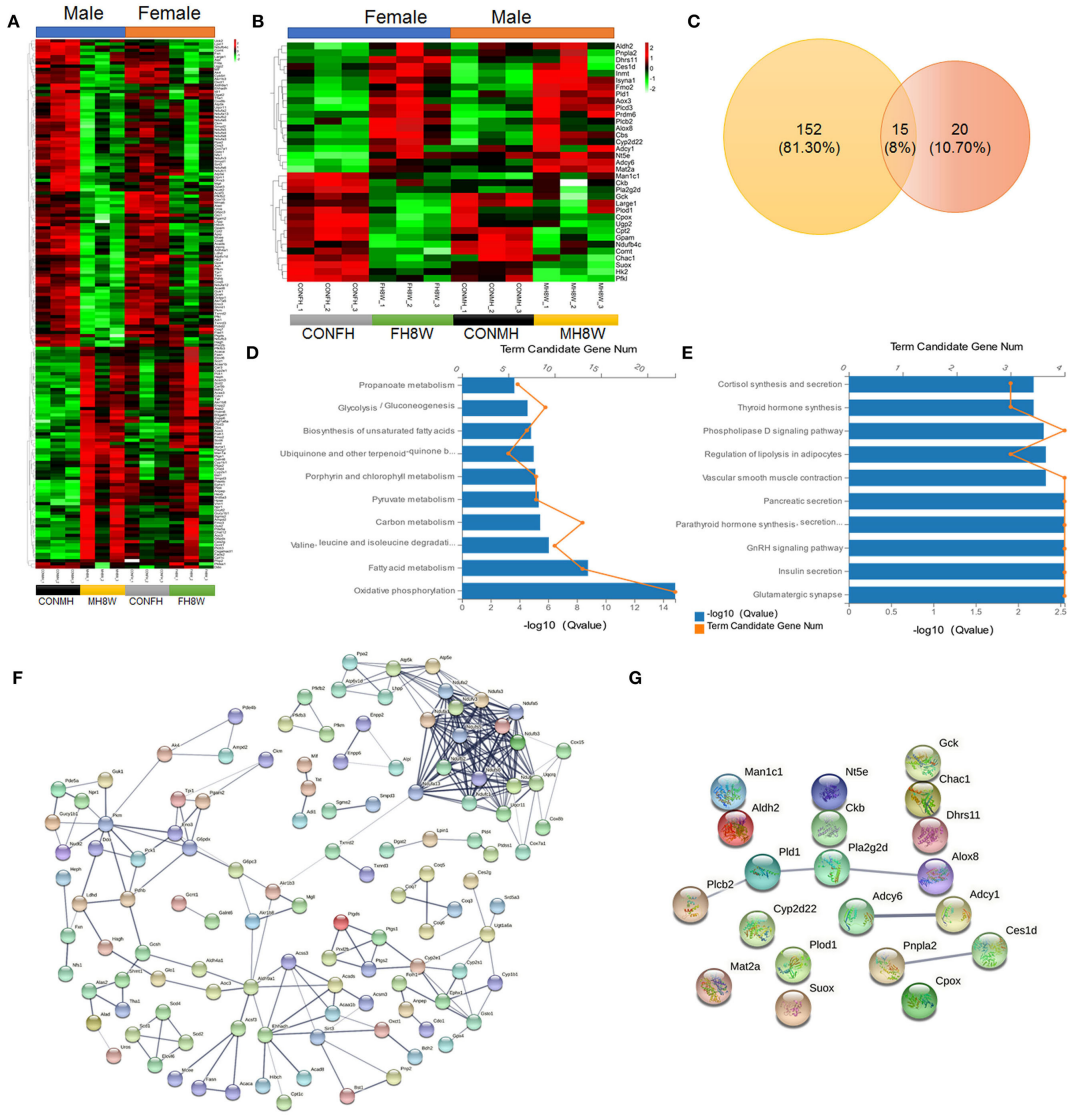
Excessive sodium intake alters the expression profile of metabolism-associated genes in mouse hearts. **(A)** Heatmaps of the metabolism-associated genes in heart tissue of male mice and the same genes were also present in female mice (*N* = 3 individual mice per group). The color bar indicates the scale used to show the expression of genes with the expression range normalized to ± 2. **(B)** Heatmaps of the metabolism-associated genes in heart tissue of female mice and the same genes were also present in male mice (*N* = 3 individual mice per group). The color bar indicates the scale used to show the expression of genes with the expression range normalized to ± 2. **(C)** Venn diagram of significantly different genes related to metabolism for HSD-treated heart tissue in male-female mice compared to the normal heart tissue in male and female mice (*N* = 3 individual mice per group). **(D)** Gene annotation of KEGG pathways enriched in metabolism-associated genes in heart tissue of HSD-treated male mice, with *P* < 0.001 (*N* = 3 individual mice per group). The top 10 pathways are shown. **(E)** Gene annotation of KEGG pathways enriched in metabolism-associated genes in heart tissue of HSD-treated female mice, with *P* < 0.05 (*N* = 3 individual mice per group). The top 10 pathways are shown. **(F,G)** The PPANs of the metabolism-associated genes in heart of male mice **(F)** and female mice **(G)** (*N* = 3 individual mice per group).

Metabolic disorders can be caused by genetic factors, high-calorie diets, and HSDs (42, 43). To investigate whether excessive sodium intake affects the metabolic processes of the heart in male and female mice, the expression of metabolism-related gene transcripts in the heart was compared between the normal and HSD groups. As shown in Figure 7A, we found that HSD had an adverse effect on the expression of 167 metabolism-related genes with significant differences in male mice, causing a serious disorder (*Q* < 0.05). Similarly, the HSD had an adverse effect on the expression of 35 metabolism-related genes with significant differences in female mice, causing a serious disorder (*Q* < 0.05) (Figure 7B). To further investigate the sex-specific effects of HSD on metabolism-related genes in mice, we compared these transcripts using Venn diagrams. As shown in Figure 7C, 152 (81.30%) genes were unique to HSD-treated male mice, and 20 (10.70%) genes were unique to HSD-treated female mice. KEGG enrichment analysis was performed to investigate the sex-specific effects of an HSD on metabolism-related pathways. For male mice, 15 metabolic pathways (*P* < 0.001) were identified, in six categories: (1) amino acid metabolism: valine, leucine, and isoleucine degradation (*Q* = 9.10E-07) and glycine, serine, and threonine metabolism (*Q* = 6.04E-04); (2) carbohydrate metabolism: pyruvate metabolism (*Q* = 4.56E-06), glycolysis/gluconeogenesis (*Q* = 2.74E-05), propanoate metabolism (*Q* = 2.25E-04), fructose and mannose metabolism (*Q* = 3.04E-04), and butanoate metabolism (*Q* = 8.03E-04); (3) energy metabolism: biosynthesis of unsaturated fatty acids (*Q* = 1.56E-05); (4) global and overview maps: oxidative phosphorylation (*Q* = 1.45E-15), fatty acid metabolism (*Q* = 1.70E-09), and carbon metabolism (*Q* = 3.65E-06); (5) lipid metabolism: biosynthesis of amino acids (*Q* = 5.97E-04) and glycerolipid metabolism (*Q* = 8.03E-04); (6) metabolism of cofactors and vitamins: porphyrin and chlorophyll metabolism (*Q* = 7.80E-06) and ubiquinone and other terpenoid-quinone biosynthesis (*Q* = 1.03E-05) (Figure 7D). For female mice, 12 metabolic pathways (*P* < 0.05) were identified, in seven categories: (1) amino acid metabolism: arginine and proline metabolism (*Q* = 2.31E-02) and lysine degradation (*Q* = 2.83E-02); (2) biosynthesis of other secondary metabolites: neomycin, kanamycin and gentamicin biosynthesis (*Q* = 3.27E-02); (3) carbohydrate metabolism: glycolysis/gluconeogenesis (HSD-treated female mice vs. HSD-treated male mice, *Q* value 3.05E-02 vs. 2.74E-05); (4) energy metabolism: sulfur metabolism (*Q* = 4.88E-02); (5) lipid metabolism: ether lipid metabolism (*Q* = 2.14E-02), glycerolipid metabolism (*Q* = 2.83E-02), steroid hormone biosynthesis (*Q* = 4.18E-02), arachidonic acid metabolism (*Q* = 4.18E-02), and glycerophospholipid metabolism (*Q* = 4.31E-02); (6) metabolism of terpenoids and polyketides: insect hormone biosynthesis (*Q* = 2.60E-02); (7) nucleotide metabolism: purine metabolism (*Q* = 1.84E-02) (Figure 7E). The STRING database was used to visualize the PPANs of these transcripts to further study the function and relevance of these metabolism-related genes. As shown in Figures 7F,G, in male mice PPANs showed a complex interaction of metabolism-related genes in different KEGG pathways (Figure 7F), but not in female mice (Figure 7G). Altogether, these results demonstrate that excessive sodium intake alters the expression profile of metabolism in the mouse heart.

### Excessive Sodium Intake Alters the Expression Profile of Immunity in Mouse Heart

**Figure 8:**
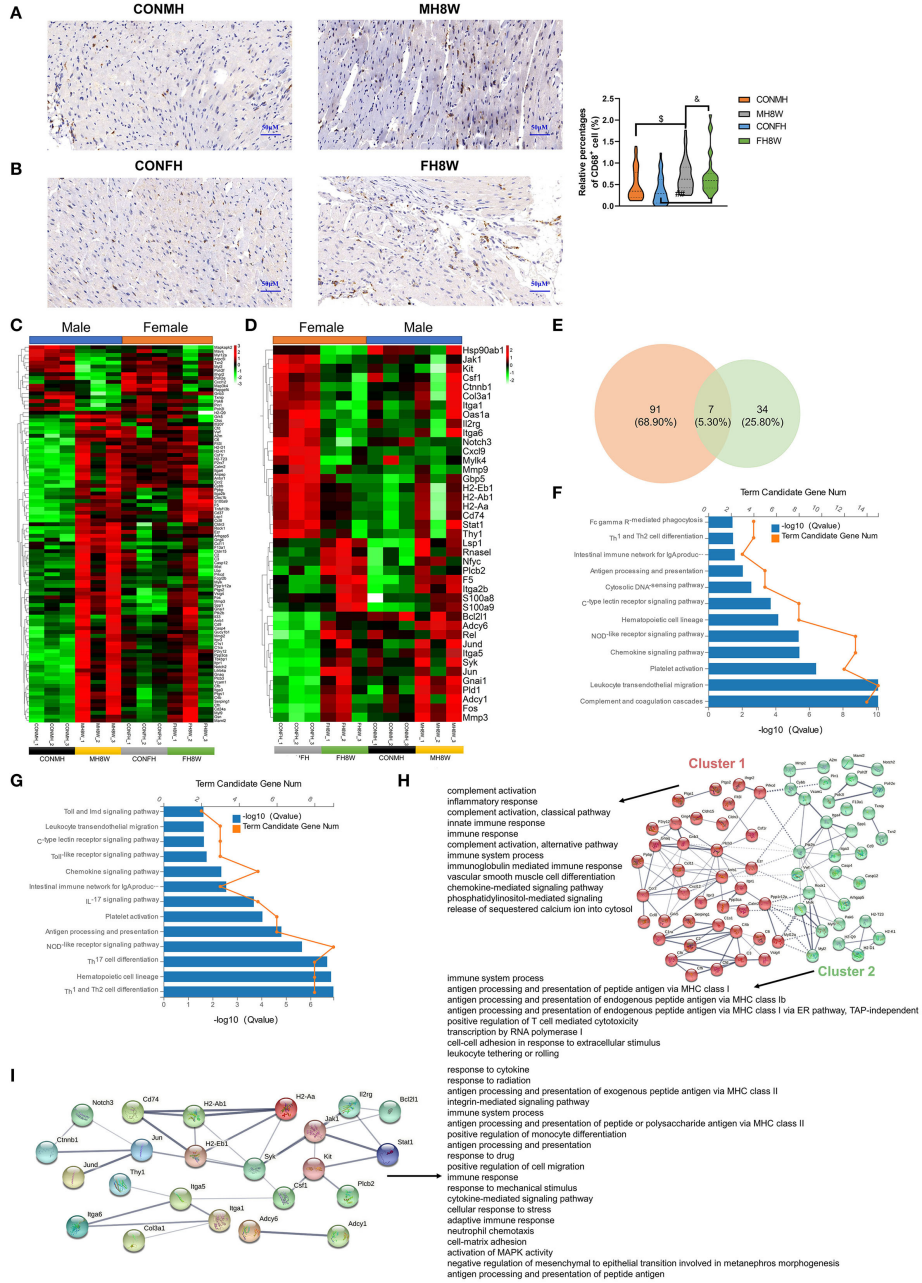
Excessive sodium intake alters the expression profile of immunity-associated genes in mouse hearts. **(A,B)** Representative immunohistochemical images (CD68^+^ macrophage) of normal heart and HSD-treated heart in male mice **(A)** and female mice **(B)** (*N* = 3 individual mice per group). The scale bar represents 50 μm. ^*$*^*P* < 0.05, ^&^*P* < 0.05, ^*##*^*P* < 0.01. **(C)** Heatmaps of the immunity-associated genes in heart of male mice and the same genes were also present in female mice (*N* = 3 individual mice per group). The color bar indicates the scale used to show the expression of genes with the expression range normalized to ± 3. **(D)** Heatmaps of the immunity-associated genes in heart of female mice and the same genes were also present in male mice (*N* = 3 individual mice per group). The color bar indicates the scale used to show the expression of genes with the expression range normalized to ± 2. **(E)** Venn diagram of significantly different genes related to immunity for HSD-treated heart tissue in male and female mice compared to the normal heart tissue in male and female mice (*N* = 3 individual mice per group). **(F)** Gene annotation of KEGG pathways enriched in immunity-associated genes in heart tissue of HSD-treated male mice, with *Q* < 0.05 (*N* = 3 individual mice per group). **(G)** Gene annotation of KEGG pathways enriched in immunity-associated genes in heart tissue of HSD-treated female mice, with *Q* < 0.05 (*N* = 3 individual mice per group). **(H)** The PPANs and biological process of the immunity-associated genes in heart tissue of male mice, with *Q* < 0.001 (*N* = 3 individual mice per group). **(I)** The PPANs and biological process of the immunity-associated genes in heart tissue of female mice, with *Q* < 0.001 (*N* = 3 individual mice per group).

High salt intake has been linked to immune cell infiltration that causes adverse effects on the heart (34, 44). To verify whether excessive sodium intake can increase macrophage infiltration in mouse heart tissue, we performed IHC to label the macrophage marker CD68 (34). As evidenced by CD68-positive cells, a two-way ANOVA demonstrated no significant sex × HSD interaction (*P* = 0.198), and macrophage infiltration was increased by HSD treatment in the hearts of male and female mice (Figures 8A,B).

To further confirm the effect of excessive sodium intake on the immunity-associated molecular signature in the hearts, we investigated the changes in immunity-associated transcript levels in the heart tissue of male and female mice. The results showed that excessive sodium intake not only alters the expression level of 98 immune-related genes in male mice but also alters the expression level of 41 immune-related genes in female mice (Figures 8C,D). Significantly different genes related to immunity in male and female mice were further compared using Venn diagrams. As shown in Figure 8E, 91 (68.90%) genes were unique to HSD-treated male mice, 34 (25.80%) genes were unique to HSD-treated female mice, and 7 (5.30%) genes were common to HSD-treated male and female mice. We further analyzed the KEGG pathways of the immune-related genes. The significant KEGG pathways of the heart in male mice (Figure 8F) and female mice (Figure 8G) were different (*Q* < 0.001). The STRING database was used to visualize the PPANs of these transcripts to further study the function and relevance of these immune-related genes. As shown in Figure 8H, PPANs are depicted based on the STRING database and are divided into two clusters with different biological processes in male mice. This result shows that these immune-related transcripts have a certain degree of complexity, and different clusters show different functional pathways. However, one cluster of these transcripts was found in female mice with 20 significant biological processes (*Q* < 0.001) (Figure 8I). Collectively, these results demonstrate that excessive sodium intake alters immune activity in the heart of male and female mice.

### Excessive Sodium Intake Alters the Expression Profile of Apoptosis in the Mouse Heart

**Figure 9:**
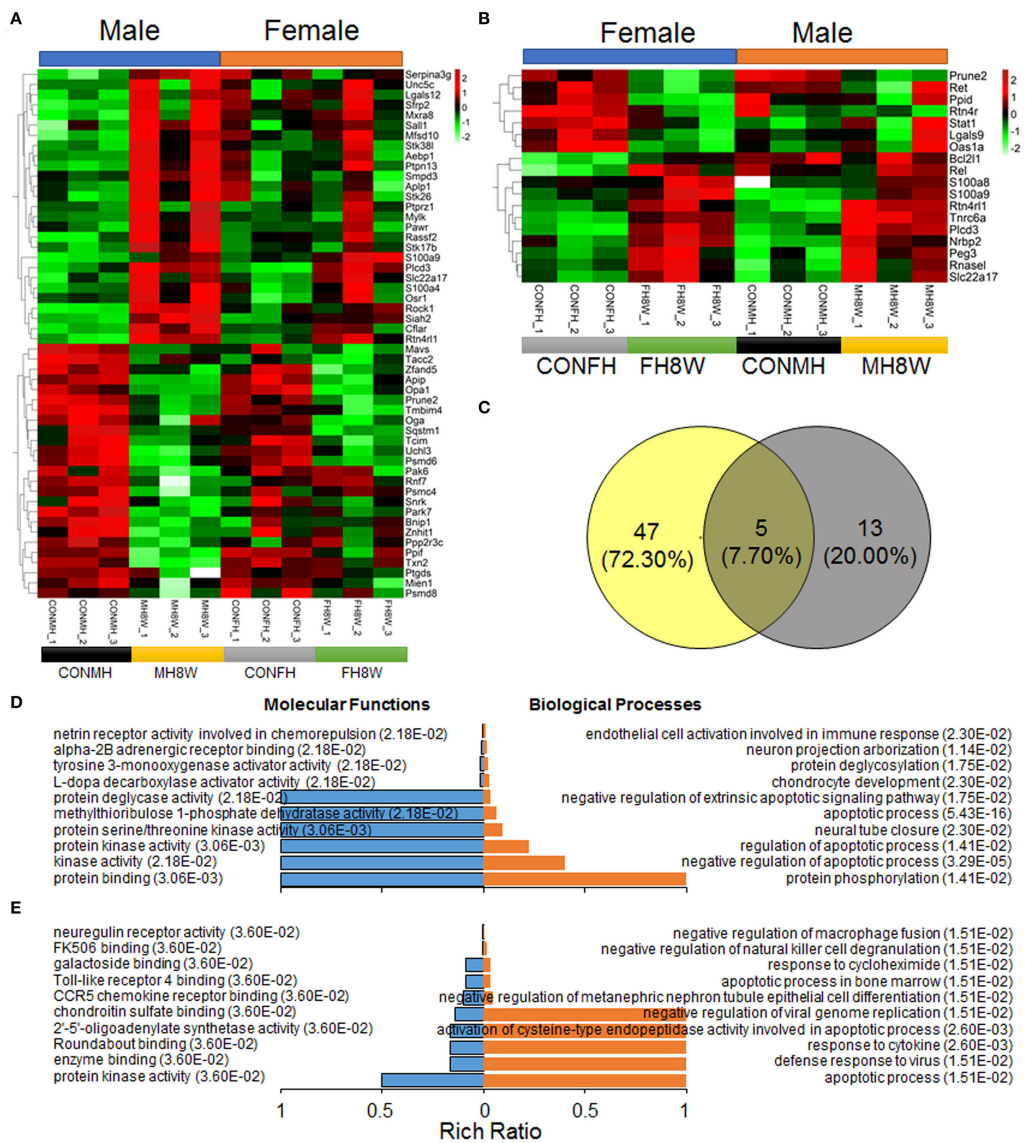
Excessive sodium intake alters the expression profile of apoptosis-associated genes in mouse heart. **(A)** Heatmaps of the apoptosis-associated gene expression in heart tissue of male mice and the same genes were also present in female mice (*N* = 3 individual mice per group). The color bar indicates the scale used to show the expression of genes with the expression range normalized to ± 2. **(B)** Heatmaps of the apoptosis-associated gene expression in heart tissue of female mice and the same genes were also present in male mice (*N* = 3 individual mice per group). The color bar indicates the scale used to show the expression of genes with the expression range normalized to ± 2. **(C)** Venn diagram of significantly different genes related to apoptosis for HSD-treated heart in male mice and female mice compared to the normal heart in male and female mice (*N* = 3 individual mice per group). **(D)** Gene annotation of molecular function and biological process enriched apoptosis-associated genes in heart tissue of male mice, with *Q* < 0.05 (*N* = 3 individual mice per group). The top 10 terms were shown. **(E)** Gene annotation of molecular function and biological process enriched apoptosis-associated genes in heart tissue of male mice, with *Q* < 0.05 (*N* = 3 individual mice per group). The top 10 terms were shown.

**Figure 10:**
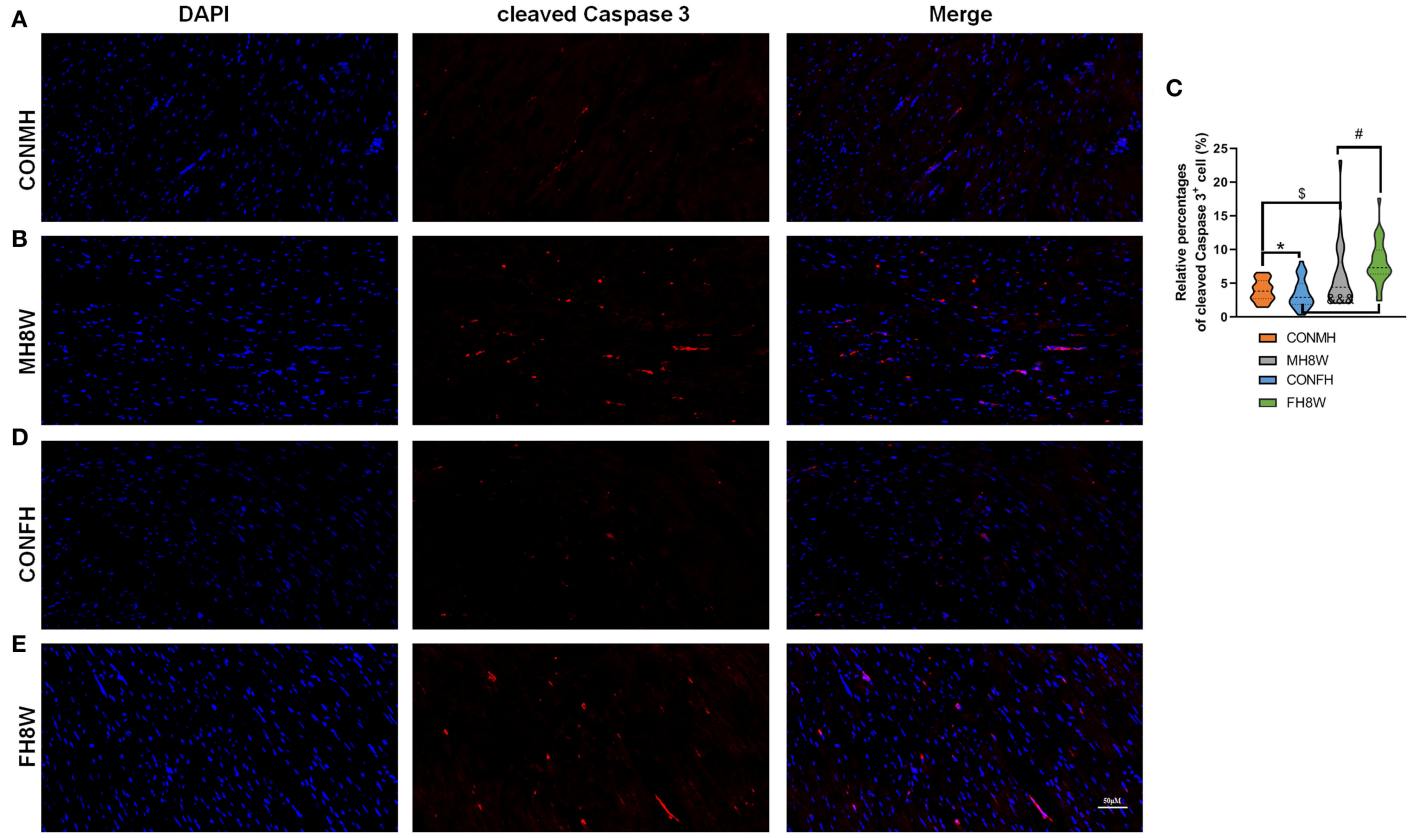
Effect of excessive sodium intake alters apoptosis of mouse heart shown by immunofluorescent staining of cleaved caspase-3. **(A,B)** Representative immunofluorescent images (cleaved caspase-3) of normal heart tissue and HSD-treated heart in male mice. The scale bar represents 50 μm. **(C)** Average relative abundance of cleaved caspase-3^+^ cells in the immunofluorescent images of cardiac sections from normal and HSD-treated heart tissue in male mice and female mice (*N* = 3 individual mice per group). ^*^P < 0.05, ^*$*^*P* < 0.05, ^#^*P* < 0.05, ^&*&&*^*P* < 0.001. **(D,E)** Representative immunofluorescent images (cleaved caspase-3) of normal and HSD-treated hearts in female mice (*N* = 3 individual mice per group). The scale bar represents 50 μm.

Studies have shown that a prenatal HSD can induce apoptosis of cardiomyocytes in the heart of offspring mice *via* the cytochrome C release pathway (45) and can aggravate the apoptosis induced by IGF-IIRα overexpression in mice (46). In this study, in order to explore whether an HSD can induce sex-specific apoptotic effects in mice, apoptosis-related gene transcripts with significant differences were analyzed (*Q* < 0.05). As shown in Figure 9, compared with mouse hearts on a normal diet, HSD-treated mouse tissue had significantly different apoptosis-related transcripts in males and females (HSD-treated male mice vs. HSD-treated female mice, 52 vs. 18) with different expression levels (Figures 9A,B). To further identify the similarities and differences in transcripts, the significantly different apoptosis-related genes in male and female mice were further compared using Venn diagrams. As shown in Figure 9C, 47 (72.30%) genes were unique to HSD-treated male mice, 13 (20.00%) genes were unique to HSD-treated female mice, and five (7.70%) genes were common to HSD-treated male and female mice. To further study the molecular functions and biological processes of these apoptosis-related genes, GO enrichment analysis was performed. The molecular functions and biological processes of these genes were changed after excessive sodium feeding in male (Figure 9D and Supplementary Tables S7, S8) and female mice (Figure 9E and Supplementary Tables S9, S10). The apoptotic process (*Q* = 5.43E-16) and activation of cysteine-type endopeptidase activity involved in the apoptotic process (*Q* = 2.60E-03) of biological process in male and female mice were identified (Figures 9D,E). To further test whether HSD can cause apoptosis in cardiac tissue, IHC analysis was performed. As shown in Figure 10, the apoptotic marker cleaved caspase-3 was present. A two-way ANOVA demonstrated a significant sex × HSD interaction (*P* = 0.032). The HSD significantly increased the expression of cleaved caspase-3 in the hearts of both male and female mice. Collectively, these results demonstrated that excessive sodium intake can induce cardiac apoptosis.

## Discussion

The meaningful finding of this study is that HSD can adversely affect the mouse heart by changing metabolism, immunity, fibrosis, and apoptosis and can induce mice to suffer from sex-specific cardiovascular disease. The conclusions of this study are mainly based on the following findings: (1) HSD has a sex-specific effect on the body mass of mice, especially on the mass of heart tissue; (2) the cardiac transcriptome is different in male and female mice; (3) the HSD-treated cardiac transcriptome showed sex-specific differences; (4) compared with the normal group, HSD produced a different number of DEGs and affects different KEGG pathways in male and female mice; (5) HSD affects the expression of genes related to fibrosis, metabolism, immunity, and apoptosis in heart tissue of male and female mice; (6) KEGG pathway enrichment analysis showed that HSD induced HCM in male mice, while HSD induced DCM in female mice. These data help us understand the negative effects of HSD on the heart in different sexes.

Existing evidence shows that sex-specific differences play an important role in the prevalence, age of onset, and/or severity of most human diseases (47), which also affects gene expression and cross-tissue genetic regulation (48). As the incidence and potential pathogenicity of diseases caused by sex-specific differences are discovered by researchers, they can be targeted for treatment in male and female patients, thereby providing more suitable options (49). The prevalence of asthma in boys is higher in childhood, the incidence of new cases in girls before and after puberty is higher (50), and genome-wide analysis reveals sex-specific gene expression in asthma patients (51). Depression and Alzheimer's disease are more common in women (52, 53), but schizophrenia, Parkinson's disease, and colorectal cancer are more common in men (54–56). Recent studies have found that mice show sexual dimorphism in response to changes in high-fat and high-salt diets. A short-term high-fat diet can lead to increased body weight, fat mass, and excessive appetite in male mice, but female mice are usually immune to the effect of short-term high-fat diet-induced changes in energy balance (57), and male *db/db* mice show higher levels of pro-inflammatory cytokines and more immune cell infiltration in the kidneys than female *db/db* mice after HSD treatment for 4 weeks (58). In this study, we also found adverse sex-specific effects on body mass, heart weight, and cell size of cardiomyocytes after HSD treatment, in accordance with previous reports (34, 48, 59).

Many human phenotypes exhibit characteristics of sex differentiation. Biological sex affects gene expression levels and cell composition throughout the human body, as has been previously reported (48, 51, 60). Consistently, we also revealed differences in the cardiac transcriptome of male and female mice. We found that in male and female mice, the number and types of highly expressed genes were different, and the number of genes unique to female mice was ~1.6 times as high as that unique to male mice. The cardiac transcriptome and number of DEGs in male mice were different from those in female mice, as revealed by PCA analysis and volcano plot, respectively. Further analysis revealed that the higher expressed genes unique to male or female mice are linked to different KEGG pathways and functional categories (normal-treated male mice vs. normal-treated male mice, 4 vs. 3).

**Figure 11:**
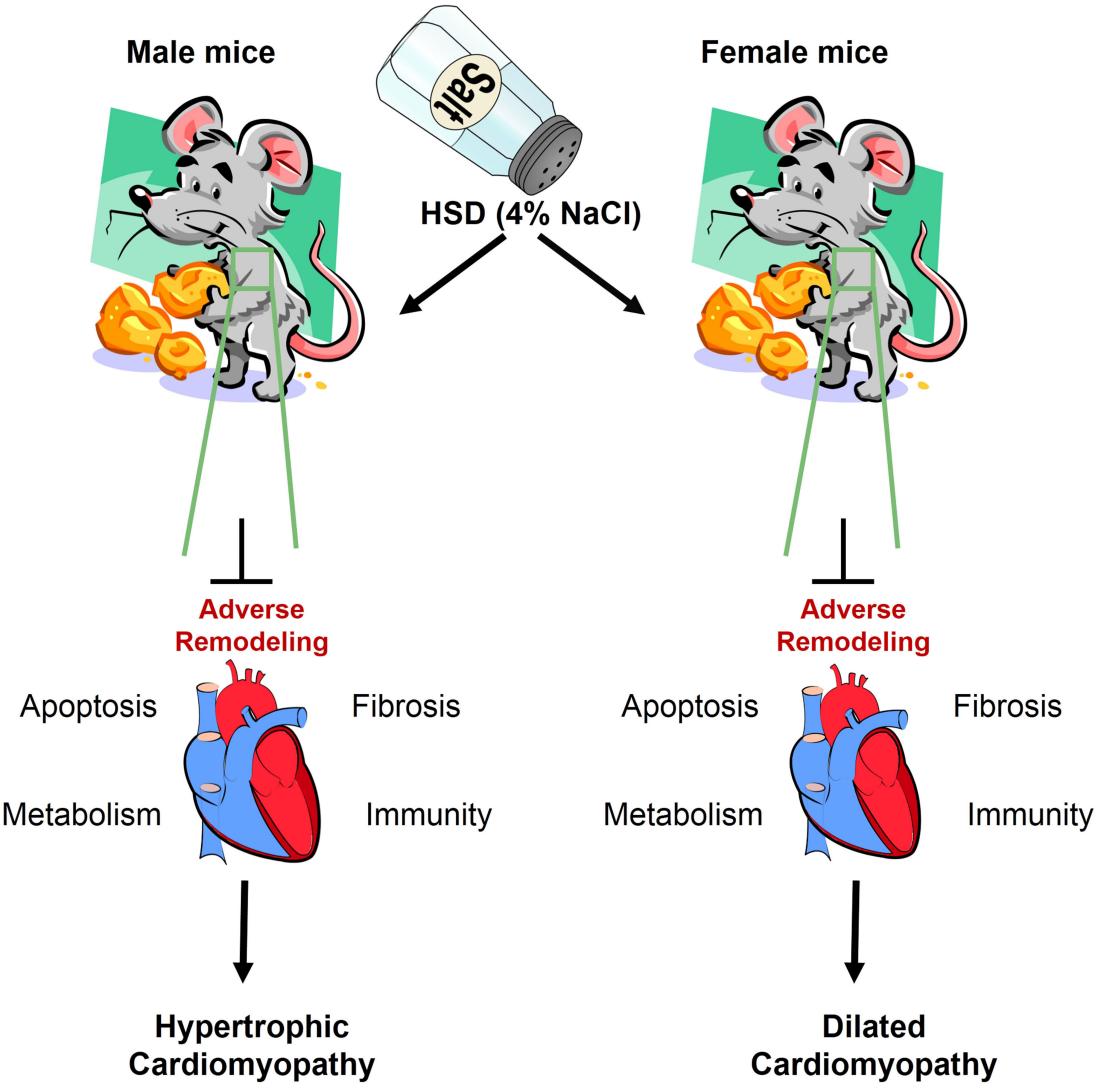
The adverse remodeling of excessive sodium intake in heart of male and female mice. HSD impairs the cardiac structure, function, and transcriptome, which results in myocardial apoptosis, cardiac fibrosis, inflammatory infiltration, and metabolic dysfunction.

In addition to the sex-specific effects mentioned above, which affect the level of gene transcription in the tissue, other factors such as HSD (61–64), high-fat diet (65–68), alcohol (69, 70), aging (71–73), and disease (74–76), change the transcriptome composition and affect the function of the tissue. Here, we focused on the impact of HSD on the cardiac transcriptome, as well as the effect of HSD on the cardiac function of mice of different sexes and the underlying molecular mechanisms. In this study, we discovered that the cardiac transcriptome was altered by HSD-treated male and female mice. After high-salt treatment in male mice, the number of genetic changes in the heart was 133 times that of female mice (HSD-treated male mice vs. HSD-treated female mice, 399 vs. 3). Further analysis revealed that the higher expressed genes unique to male or female mice are linked to different KEGG pathways and different categories of biological function (normal-treated male mice vs. normal-treated female mice, 11 vs. 20). Several studies have highlighted the harmful effects of HSD on tissue function. HSD has been associated with hypertension (77, 78), cardiovascular disease (79, 80), erectile dysfunction (81, 82), and obesity (63, 79). Here, we also show that HSD can induce HCM in male mice and DCM in female mice by altering the expression of fibrosis-, metabolism-, immunity-, and apoptosis-related genes (Figure 11). Therefore, HSD alters the sex-specific transcriptomic profile of the murine heart.

Fibrosis is a recognized cause of morbidity and mortality (83). Myocardial fibrous scars are most commonly caused after myocardial infarction (84). However, various other diseases promote myocardial fibrosis, such as hypertensive heart disease, diabetic hypertrophic cardiomyopathy, and idiopathic dilated cardiomyopathy (84, 85). Fibrosis is a pathological process of extracellular matrix remodeling, resulting in abnormal matrix composition and quality, and impaired myocardial function (84). Studies have shown that HSD can induce cardiac hypertrophy and fibrosis in male mice (86, 87). In this study, we also demonstrated that HSD significantly accelerated the formation of cardiac fibrosis and dramatically changed the expression profile of fibrosis, including composition, molecular function, and biological process, in both male and female mice.

High-salt-induced hypertension, renal disease, and related metabolic disorders are thought to be disorders caused by the interaction of genetic background and dietary factors such as HSD, although the exact mechanism is still unclear (88, 89). Changes in certain metabolites can affect the entire metabolic network and state of the organism through interactions with other metabolites (90). Existing studies have shown that HSD greatly changes the metabolic activities of the heart (91, 92), plasma (88, 93), muscle (88), liver (91, 94), and kidney (89). Herein, we demonstrated that the expression profile of metabolism-associated genes, KEGG pathways, and PPANs of the heart were affected by HSD treatment in male and female mice.

Recent studies have shown that HSD also has direct and indirect effects on immune cells and is considered a potential regulator of inflammation and autoimmune diseases, leading to an overall imbalance in immune homeostasis (2, 95, 96). In Dahl SS rats with salt-sensitive hypertension, immune cells in the kidney, especially macrophages and lymphocytes, infiltrate and are linked to kidney damage caused by HSD (97, 98). Kataoka et al. (34) also showed that HSD can increase the infiltration of CD45^+^ and CD68^+^ inflammatory cells in the heart of male mice. In this study, we demonstrated that HSD increased macrophage infiltration in both male and female mice. HSD dramatically changed the expression profile of immunity and associated KEGG pathways, PPANs, and biological processes in the hearts of both male and female mice. This is consistent with previous studies, which have shown that HSD affects the innate immune system of macrophages, primarily by causing changes in the activation state of macrophages (99) and promotes the differentiation of Th1 and Th17 cells as well as the expression of inflammatory factors and genes (100). Therefore, HSD is considered to be one of the main factors of environmental-induced immune disorders.

Apoptosis plays a key role in the pathogenesis of hypertrophied, infarcted, senescent, and failing hearts, and HSD is related to cardiac cell proliferation and apoptosis (34). Herein, we discovered that HSD affected the expression of apoptosis-related genes in the mouse heart and that HSD had more impact on apoptosis-related genes in the heart of male mice than in female mice. GO analysis of enriched apoptosis-associated genes in the heart tissue of male mice revealed that HSD significantly activates apoptotic processes and other cellular processes in the heart tissue of male and female mice. This is consistent with maternal high-salt intake-induced cardiomyocyte apoptosis in the adult offspring's heart. HSD also activates pro-apoptotic and pro-inflammatory signaling pathways in the retina (101). However, the mechanism of HSD-induced effects on cardiomyocyte apoptosis needs to be further explored.

Although our data have shown the harmful effects of HSD on mouse cardiac gene expression, this study has several limitations. First, this study was only carried out in C57BL/6J mice administered an HSD with 4% NaCl for 8 weeks. The harmful effects on the heart need to be further explored by adopting higher salt concentrations, longer diet cycles, and a model of senescence. Second, this research focuses on transcriptome research; therefore, the effects of an HSD on metabolites and proteomics need to be investigated. Finally, the mouse model used in this study is nocturnal, which is a key behavioral difference to humans. Therefore, the clinical guiding significance of the conclusions of this study needs to be viewed dialectically.

In summary, our results have demonstrated the mechanism of HSD-induced heart damage leading to cardiovascular disease from the perspective of transcriptome analysis and provide a theoretical basis for potential sex-specific clinical risk assessment, pathogenic mechanisms, and interventions for the treatment of cardiac damage.

## Data Availability Statement

The datasets presented in this study can be found in online repositories. The names of the repository/repositories and accession number(s) can be found in the article/Supplementary Material. The raw data have been deposited into sequence read archive (SRA) database: https://www.ncbi.nlm.nih.gov/bioproject/PRJNA788158.

## Ethics Statement

The animal study was reviewed and approved by Henan Province People's Hospital Institutional Animal Care and Use Committee.

## Author Contributions

SH, XC, and HW: conceptualization, funding, resources, and supervision. SH and XC: methodology, investigation, analysis, visualization, and writing—review and editing. All authors contributed to the article and approved the submitted version.

## Funding

Research support was provided by the National Natural Science Foundation of China (Grant Number 82101089 to SH), Natural Science Foundation of Henan Province (Grant Number 222300420361 to XC), the Key R&D and Promotion Special Program of Henan Province (Grant Numbers 212102311011 and 222102310120 to SH and 212102310477 to HW), the Henan Provincial Medical Science and Technology Research Joint Co-construction Project (Grant Numbers LHGJ20200064 to SH and SB201901087 to HW), the Basic Science Project for Youth of Henan Eye Institute/Henan Eye Hospital (Grant Number 20JCQN003 to SH), and the Doctoral Research and Development Foundation of Henan Provincial People's Hospital (Grant Numbers ZC20190146 to SH and ZC20200230 to XC).

## Conflict of Interest

The authors declare that the research was conducted in the absence of any commercial or financial relationships that could be construed as a potential conflict of interest.

## Publisher's Note

All claims expressed in this article are solely those of the authors and do not necessarily represent those of their affiliated organizations, or those of the publisher, the editors and the reviewers. Any product that may be evaluated in this article, or claim that may be made by its manufacturer, is not guaranteed or endorsed by the publisher.
